# Crystal structure of the cytotoxic macrocyclic trichothecene Isororidin A

**DOI:** 10.1107/S2053229624006144

**Published:** 2024-07-10

**Authors:** Mostafa A. Asmaey, Dimitris A. Kalofolias, Maria-Despoina Charavgi, Ismail R. Abdel-Rahim, Evangelia D. Chrysina, Dennis Abatis

**Affiliations:** a Division of Pharmacognosy and Natural Products Chemistry, Department of Pharmacy, School of Health Sciences, National and Kapodistrian University of Athens, Athens 15771, Greece; b Department of Chemistry Faculty of Science Al-Azhar University Assiut branch Assiut 71524 Egypt; c Institute of Chemical Biology, National Hellenic Research Foundation, 48 Vassileos Constantinou Avenue, Athens 11635, Greece; d Botany and Microbiology Department, Faculty of Science, Assiut University, Egypt; University of Melbourne, Australia

**Keywords:** crystal structure, Isororidin A, Roridin A, macrocyclic trichothecenes, *Myrothesium verrucaria*, roridoid, mycotoxin

## Abstract

The highly cytotoxic macrocyclic trichothecene Isororidin A (C_29_H_40_O_9_) was isolated from the fungus *Myrothesium verrucaria* endophytic on the wild medicinal plant ‘Datura’ (*Datura stramonium* L.) and was characterized by one- (1D) and two-dimensional (2D) NMR spectroscopy. The three-dimensional structure of Isororidin A has been confirmed by X-ray crystallography at 0.81 Å resolution from crystals grown in the ortho­rhom­bic space group *P*2_1_2_1_2_1_, with one mol­ecule per asymmetric unit. Isororidin A is the epimer of previously described (by X-ray crystallography) Roridin A at position C-13′ of the macrocyclic ring.

## Abstract 2

The absolute configuration of Isororidin A, isolated from the fungus *Myrothesium verrucaria*, has been determined by X-ray crystallography.

## Introduction

Macrocyclic trichothecenes (MTs) constitute the second major group (the other being the simple trichothecenes) of a class of highly functionalized sesquiterpenoid secondary metabolites, mainly of fungal origin, which are well known for their severe toxicity to both animals and humans (Grove, 2007 ▸; Shank *et al.*, 2011 ▸; Wu *et al.*, 2017 ▸). Most trichothecenes are at least tetra­cyclic, as they contain a spiro-epoxide group in the 12–13 position of the ‘trichothecane’ sesquiterpene skeleton. They also usually com­prise a double bond at C9—C10; thus, they are considered as 12–13 ep­oxy-trichothec-9-ene derivatives [see (*a*) in Scheme 1]. In MTs, an extra cyclic diester or triester ring is connected to the trichothecene core skeleton at C-4 and C-15, making them penta­cyclic macrolides. The presence of the spiro-ep­oxy group, the Δ^9,10^ bond and the macrocyclic ring in the mol­ecule appear to be crucial for their biological properties, which include anti­fungal, anti­malarial, anti­virus and anti­cancer activity (de Carvalho *et al.*, 2015 ▸; Jarvis & Mazzola, 1982 ▸; McCormick *et al.*, 2011 ▸; Wu *et al.*, 2017 ▸). The MTs are further classified into the subgroups of the Roridoids, to which Roridin A and Isororidin A belong [see (*b*) and (*c*) in Scheme 1, respectively], the Baccharinoids, the Verrucaroids and the Trichoverroids, which are considered the biosynthetic precursors of the three former subgroups of MTs (Bräse *et al.*, 2009 ▸).

There has been a series of articles since the 1980s con­cer­ning the elucidation of the configuration of the stereogenic centres of the macrocyclic ring of the MTs, especially C-6′ and C-13′ in the Roridoids (Jarvis *et al.*, 1982 ▸, 1987 ▸, 1996 ▸; Jarvis & Wang, 1999 ▸). The task was based mainly on NMR spectroscopy (despite the technical limitations of the method at that time), as well as chemical manipulations when there were adequate qu­anti­ties available, aided – in rare cases – by stereoselective synthesis and X-ray diffraction analyses. In 1982, Jarvis and co-workers isolated Roridin A and Isororidin A from a large-scale fermentation of *Myrothesium verrucaria* and resolved the relative configuration of Roridin A by X-ray crystallography. The absolute configuration of Roridin A was confirmed after oxidative cleavage of its hy­droxy­ethyl moiety, which produced Verrucarin A, an MT whose absolute con­fig­uration had already been established (Jarvis *et al.*, 1982 ▸). The ^1^H and ^13^C NMR spectra of Roridin A and Isororidin A in CDCl_3_ were almost identical, except for carbon C-6′, which differed in the ^13^C NMR spectra by 1.1 ppm. The epimeric relation of the two fungal metabolites at C-13′ was deduced indirectly by the selective hydrogenation of Roridin A and Isororidin A to their respective tetra­hydro derivatives, and then oxidation of the C-6′ hy­droxy­ethyl group of these tetra­­hydro derivatives to an identical (in the ^1^H NMR spectrum) corresponding methyl ketone (Jarvis *et al.*, 1982 ▸). Even though Isororidin A was re-isolated a few times in subsequent years from different fungal strains and by different research groups, verification of its structure was performed only by com­parison of the NMR data in CDCl_3_ with those reported in 1982, but without submitting the NMR data. Isororidin A is one of the most cytotoxic metabolites among all com­pounds containing C, H and O, and was on the shortlist of the National Cancer Institute (NCI) for the most promising anti­cancer agents in the 2000s (Amagata *et al.*, 2003 ▸; de Carvalho *et al.*, 2015 ▸; Sy-Cordero *et al.*, 2010 ▸). The mechanism of action of the macrocyclic trichothecenes is still underexplored, possibly due to their severe general cytotoxicity. However, there is evi­dence that MTs show large variations in both activity and selectivity against different cancer cell lines induced by alterations in their mol­ecular structure. These findings indicate that MTs may still be considered as highly promising anti­tumour agents, as long as more detailed structure–activity relationship (SAR) and qu­anti­tative structure–activity relationship (QSAR) studies have been performed. For these studies, knowledge of the configuration and conformation of the MTs is undoubtedly critical (Wu *et al.*, 2017 ▸; Zhu *et al.*, 2020 ▸). In the current article, Isororidin A was isolated from the fun­gus *Myrothesium verrucaria* endophytic on the wild medicinal plant ‘Datura’ (*Datura stramonium* L.) and was characterized by 1D and 2D NMR spectroscopy. Its crystal structure is presented for the first time at 0.81 Å resolution.

## Experimental

### Isolation and crystallization

Isororidin A was isolated as a colourless solid (19.5 mg) after high-performance liquid chromatography (HPLC) using a semipreparative C18 column eluted with a linear gradient mixture of water and methanol. The gross structure of the com­pound was elucidated on the basis of a detailed analysis of its 1D/2D NMR and high-resolution mass spectroscopic (HRMS) data. The full 1D and 2D NMR data recorded in CD_3_OD are reported for the first time (see the *Analytical data* section in the supporting information and Table 1 ▸). The relative configuration of its chiral centres was deduced from a combined study of nuclear Overhauser effect (NOE) correlations and ^3^*J*_HH_ coupling constants, and by com­parison with the NMR data of other Roridoids having similar structures (Amagata *et al.*, 2003 ▸; Jarvis & Wang, 1999 ▸). The absolute configuration of all its chiral centres was confirmed by the X-ray crystallographic analysis of its colourless needle-like crystals that were obtained after the slow evaporation of a solution in methanol from an NMR tube. Most of the Isororidin A crystals had morphological defects that may have led to twinned spots on the diffraction pattern and potential issues at the stage of processing and deconvolution. Therefore, a small fragment of an Isororidin A crystal, with the least morphological defects, was isolated and mounted on a litho loop to minimize the background contribution when exposed to X-rays. The loop was placed on the goniometer head and diffraction data were collected at 0.81 Å resolution.
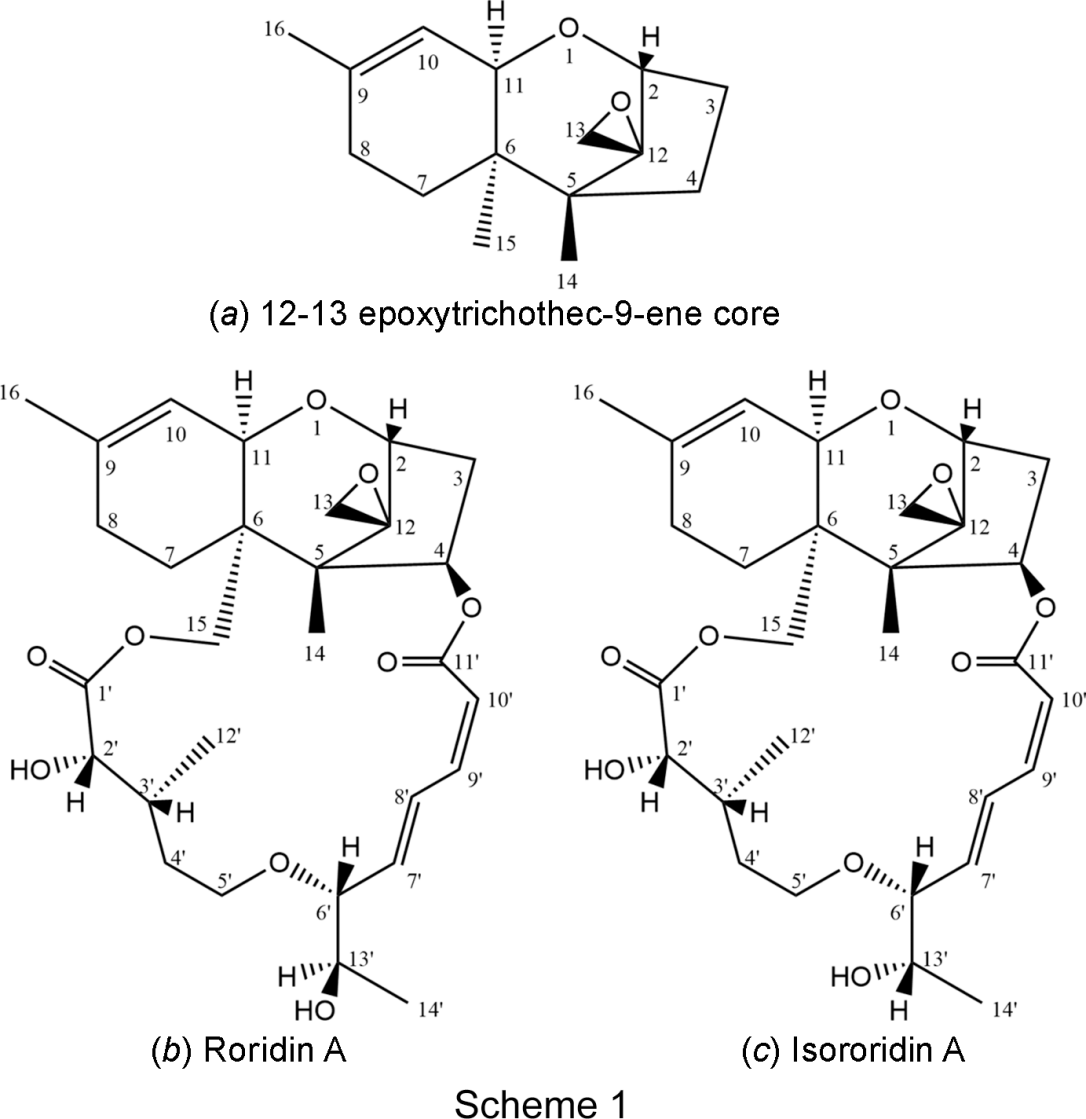


### Refinement

Crystal data, data collection and structure refinement details are summarized in Table 2 ▸. Details of the geometry of the Isororidin A crystal structure regarding bond lengths (Å), bond angles (°), torsion angles (°) and the geometry of the hydrogen bonds [distances (Å) and angles (°)] are presented in the supporting information (Tables S1–S5).

## Results and discussion

### Structural commentary

The crystal structure of Isororidin A, isolated from the ethyl acetate extract of the culture broth of the endophytic fungus *M. verrucaria*, after a series of chromatographic separations, is presented at 0.81 Å resolution and confirms the configuration at position C13′. Isororidin A crystallized in the ortho­rhom­bic space group *P*2_1_2_1_2_1_ (No. 19). A data set was initially collected at room temperature at 0.81 Å resolution (Table S1 in the supporting information) and the calculated Flack parameter (Flack, 1983 ▸; Parsons *et al.*, 2013 ▸) was 0.4 (4), which was not sufficient to assess the absolute configuration of Isororidin A. Therefore, a new data set was collected at 100 K. The crystal lattice and space group remained *P*2_1_2_1_2_1_, with unit-cell dimensions *a* = 9.2707 (4), *b* = 15.2236 (6), *c* = 20.0806 (8) Å and α = β = γ = 90°, and the Flack parameter calculated for this structure was −0.02 (3), confirming the absolute configuration of Isororidin A. The experimental details are summarized in Table 2 ▸ and Tables S2–S6 of the supporting information. The two experiments reveal no temperature-dependent phase change, as the unit-cell param­eters are almost identical (Table 2 ▸ and Table S1). The measurement at 100 K resulted in an overall better data set with an improved *R* parameter and a higher precision Flack parameter. Therefore, the structure analysis that follows focuses on the structure determined at 100 K.

**Figure 1:**
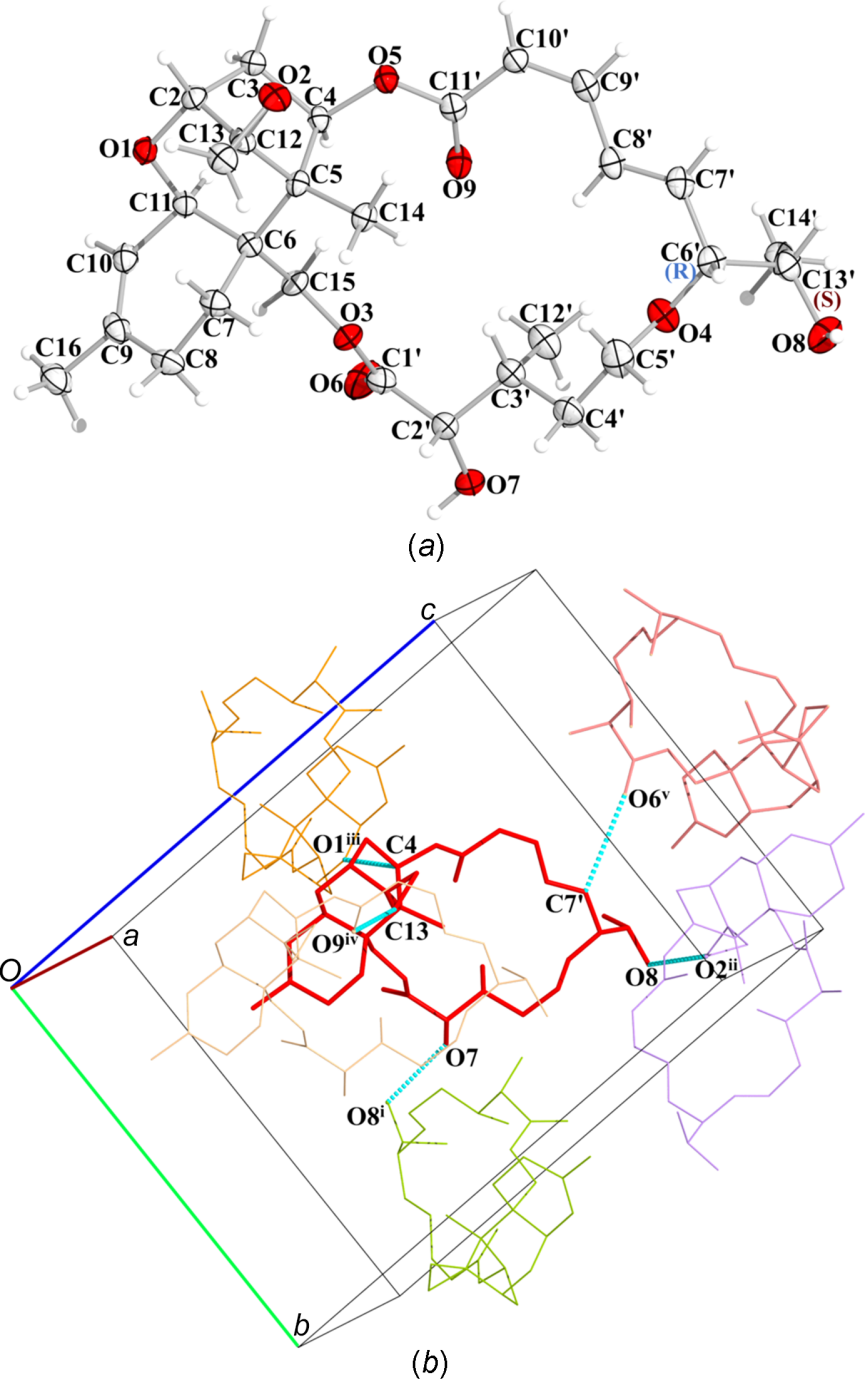
(*a*) Schematic representation of the Isororidin A X-ray diffraction solution, drawn with 50% probability displacement ellipsoids. O atoms are shown in red, C atoms in light grey and H atoms in pale pink. The absolute configurations of C6′ (*R*) and C13′ (*S*) shown in Scheme 1 are indicated. (*b*) A view of the inter­molecular hydrogen-bond inter­actions formed between Isororidin A (shown in red) and its symmetry-related mol­ecules [colour code for symmetry codes: (*x* + 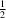, −*y* + 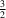, −*z* + 1) in lime, (−*x* + 1, *y* + 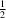, −*z* + 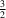) in lavender, (*x* − 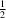, −*y* + 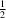, −*z* + 1) in orange, (*x* + 1, *y*, *z*) in tan and (−*x* + 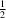, −*y* + 1, *z* + 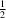) in salmon], while the hydrogen bonding is indicated with blue dashed lines.

The packing of the mol­ecules is stabilized by two inter­molecular hydrogen-bond inter­actions between atom O7, which acts as a donor to symmetry-related O8^i^ and O8, which acts as a donor to symmetry-related O2^ii^, as well as inter­molecular C—H⋯O inter­actions between C4 and O1^iii^, C13 and O9^iv^, and C7′ and O6^v^ (see Table 3 ▸ for symmetry codes). A schematic representation of the crystal structure, showing the stereoconfiguration of Isororidin A and its packing within the unit cell, is presented in Fig. 1 ▸.

Superposition of the crystal structure of Isororidin A with the only available previously determined structure of Roridin A (CCDC deposition No. 1110357, CSD refcode BIDPIN10; Jarvis *et al.*, 1982 ▸) showed that the overall structure is the same; more pro­nounced differences are observed in the macrocyclic ring, more specifically, in the vicinity of the C13′ atom [Figs. 1 ▸(*a*) and 2 ▸]. Both saturated pyran rings adopt dis­torted chair con­formations, with a torsion angle C5—C6—C11—O1 of −46.7 (2)° in Isororidin A *versus* −41.5° in Roridin A. The un­saturated cyclo­hexene rings adopt flattened half-chair con­formations, while the five-membered rings in both structures adopt envelope conformations, with the C atom at position C12 (C11 for the Roridin A structure) pointing out of the plane. The differences observed between the two structures relate to the hy­droxy­ethyl group and neighbouring atoms that include a significant rotation of the torsion angles O4—C6′—C13′—O8 and C7′—C6′—C13′—O8 by 106.7 and 104.6°, respectively. Additional differences are observed for torsion angles O7—C2′—C3′—C4′ by 18.4°, O7—C2′—C3′—C12′ by 17.2°, O9—C11′—C10′—C9′ by 17.7°, O5—C11′—C10′—C9′ by 16.2°, C7′—C6′—C13′—C14′ by 10.9°, O4—C6′—C13′—C14′ by 9.2° and O6—C1′—C2′—C3′ by 7.3°. The rest of the differences in the torsion angles ob­served in the 18-membered macrocyclic ring are less profound and in the range of 5°; for example, torsion angle O6—C1′—C2′—O7′ by 4.2° (Table S7 in the supporting information). These changes may be attributed to the inter­molecular inter­actions formed in Isororidin A com­pared to Roridin A [Fig. 1 ▸(*b*)].

### Supra­molecular features

**Figure 3:**
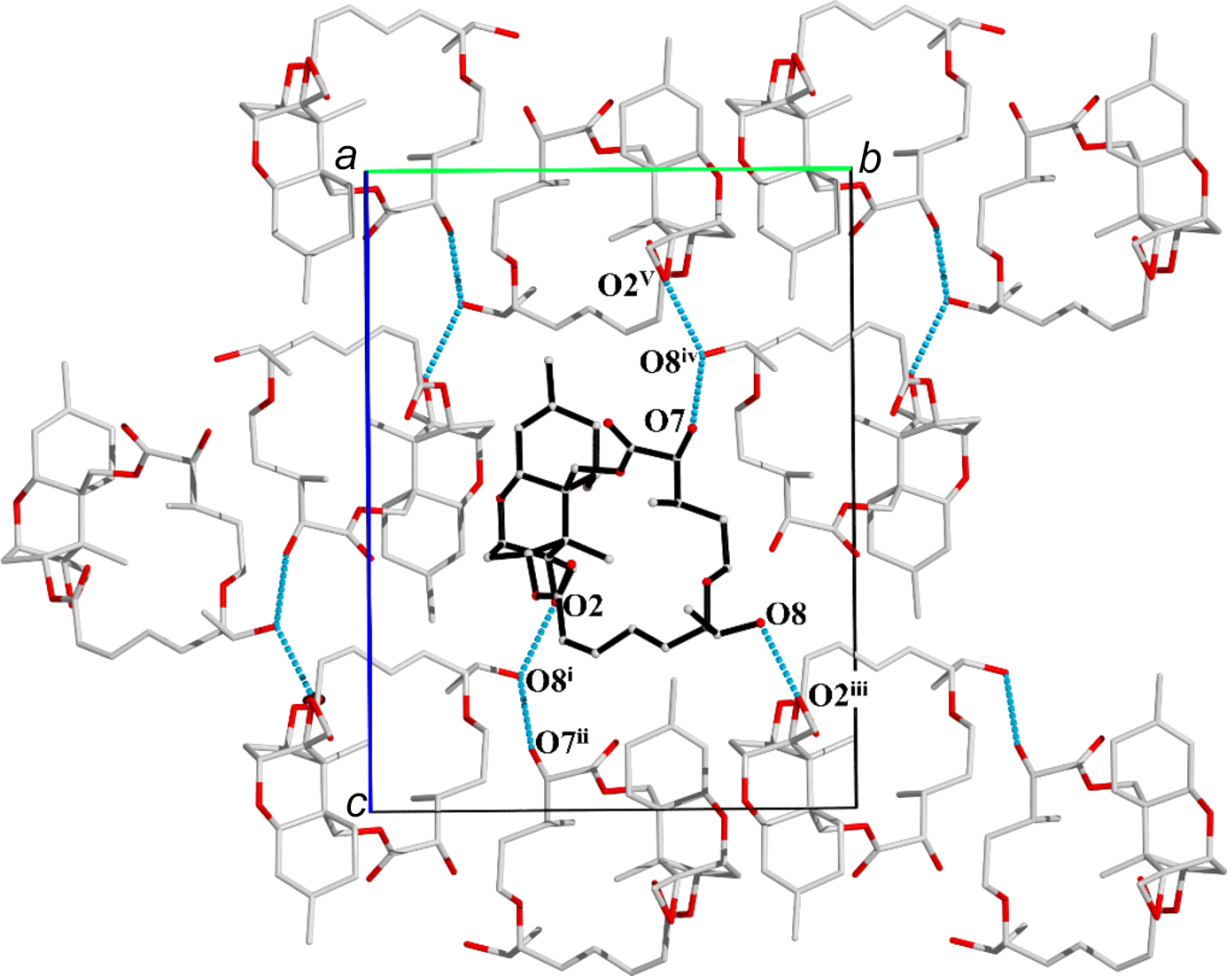
Schematic representation of the supra­molecular structure of Isororidin A. The asymmetric unit is highlighted in black and the hydrogen bonds are indicated in blue. [Symmetry codes: (i) −*x*+, *y* − 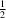, −*z* + 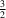; (ii) −*x* + 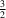, −*y* + 1, *z* + 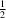; (iii) −*x* + 1, *y* + 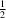, −*z* + 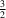; (iv) −*x* + 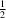, −*y* + 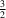, −*z* + 1; (v) −*x* + 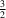, −*y* + 1, *z* − 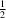.]

A schematic representation of the structure of Isororidin A and its packing with symmetry-related mol­ecules within the crystal is shown in Fig. 3 ▸. Isororidin A crystallized in the ortho­rhom­bic space group *P*2_1_2_1_2_1_. The difference observed in the epimeric C atom seems to foster the inter­molecular inter­actions within the unit cell. Atom O8 is hydrogen bonded to O2 of a symmetry-related mol­ecule within the unit cell, while in the case of Roridin A, the same atom inter­acts with O1.

### Database survey

One entry is available in the Cambridge Structural Database (CSD; Groom *et al.*, 2016 ▸) for the structure of Roridin A (CCDC deposition No. 1110357, CSD refcode BIDPIN10; Jarvis *et al.*, 1982 ▸) determined in the space group *P*2_1_ with unit-cell dimensions *a* = 10.197 (3), *b* = 14.079 (4), *c* = 9.606 (2) Å, α = γ = 90° and β = 94.6 (1)°.

## Supplementary Material

Crystal structure: contains datablock(s) I, II, global. DOI: 10.1107/S2053229624006144/ux3006sup1.cif

Structure factors: contains datablock(s) I. DOI: 10.1107/S2053229624006144/ux3006Isup2.hkl

Supporting information file. DOI: 10.1107/S2053229624006144/ux3006Isup3.cdx

Additional data for the room-temperature determination. DOI: 10.1107/S2053229624006144/ux3006sup4.pdf

Structure factors: contains datablock(s) II. DOI: 10.1107/S2053229624006144/ux3006IIsup5.hkl

CCDC references: 2083096, 2279458

## Figures and Tables

**Figure 2:**
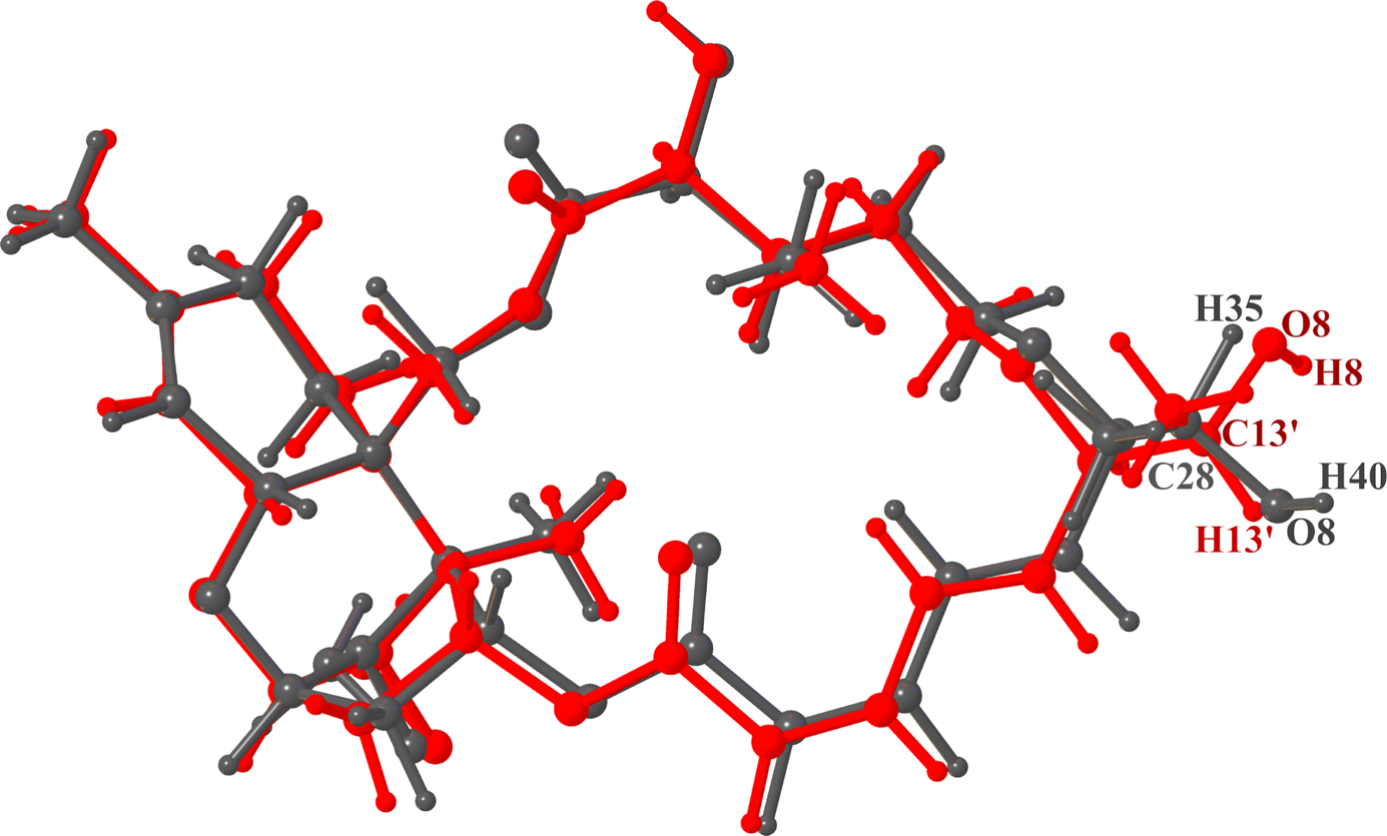
Superposition of the three-dimensional structures of determined Isororidin A (with an *S* configuration at C13′) and its stereoisomer (epimeric at C28 with an *R* configuration) Roridin A. Isororidin A is shown in red and Roridin A in grey.

**Table 1:** NMR spectroscopic data for Isororidin A [400 (^1^H) and 100 MHz (^13^C), δ ppm]*^*a*^*

Position (Scheme 1)	^1^H NMR (*J* in Hz)	^13^C NMR	COSY	HMBC	NOESY
2	3.74 (*d*, 5.1)	80.4	3b	4, 5, 12	3′, 13a
3	b: 2.14 (overlap by H-3′)	35.7	2, 4	2, 4	
	a: 2.47 (*dd*, 8.2, 15.2)			2, 5, 12	
4	5.84 (*dd*, 4.5, 8.2)	76.0	3	2, 3, 5, 6, 12, 11′	11
5	Cq	50.5			
6	Cq	45.0			
7	1.87 (*m*, 2H)	21.3	8	6, 8, 9, 11	13, 14
8	a: 1.93 (*d*, 8.0)	28.7	7	6,7, 9, 10	
	b: 1.98 (*m*)				
9	Cq	141.7			
10	5.41 (*d*, 5.4)	119.7	11, 16	6, 8, 11, 16	
11	3.72 (*br d*, 5.4)	68.5	10	7, 10, 15	4
12	Cq	66.4			
13	2.86 (*d*, 4.0)	48.5		2, 5, 12	14
	3.05 (*d*, 4.0)				
14	0.81 (*s*)	8.0	,	4, 5, 6, 12	2′, 3′, 15, 12′
15	4.32 (*d*, 12.2)	64.8	15	5, 6, 7, 1′	14
	4.46 (*d*, 12.2)			5, 6, 7, 11, 1′	
16	1.72 (*s*)	23.3	10	8, 9, 10	
1′	CO	175.6			
2′	4.04 (*d*, 4.0)	76.7	3′	1′, 4′, 12′	14, 3′, 12′
3′	2.08 (*m*	37.7	2′, 12′	1′, 2′	14, 2′
4′	1.58 (*m*)	34.9	4′, 5′	3′, 5′, 12′	
	1.73 (*m*)			2′, 3′, 5′	
5′	3.50 (*ddd*, 5.2, 8.7, 9.1)	70.9	4′, 5′	3′, 4′, 6′	
	3.58 (*ddd*, 5.2, 9.6, 9.8)				
6′	3.82 (*m*)	84.6	7′, 13′	5′, 7′, 8′, 14′	8′, 14′
7′	6.17 (*dd*, 3.0, 15.4)	142.3	6′, 8′	6′, 8′, 9′	13′, 14′
8′	7.60 (*ddt*, 11.4, 15.4, 1.1)	126.8	7′, 9′	6′, 9′, 10′	14, 3′, 10′, 12′
9′	6.75 (*t*, 11.4)	145.5	8′, 10′	7′, 8′, 11′	7′
10′	5.76 (*d*, 11.2)	117.9	9′	8′, 9′, 11′	14
11′	CO	168.1			
12′	1.09 (*d*, 6.8)	15.1	3′	2′, 3′, 4′	14, 2′, 3′, 8′
13′	3.69 (*m*)	71.0	6′, 14′	6′, 14′	7′, 8′, 14′
14′	1.16 (*d*, 6.4)	18.4	13′	6′, 13′	6′, 7′, 8′,13′

Note: (*a*) the assignments were based on ^1^H–^1^H COSY, HSQC–DEPT and HMBC experiments, and recorded in MeOD-*d*_4_.

**Table 2:** Experimental details

Crystal data	
Chemical formula	C_29_H_40_O_9_
*M*_r_	532.61
Crystal system, space group	Orthorhombic, *P*2_1_2_1_2_1_
Temperature (K)	100
*a*, *b*, *c* (Å)	9.2707 (4), 15.2236 (6), 20.0806 (8)
*V* (Å^3^)	2834.0 (2)
*Z*	4
Radiation type	Cu *K*α
μ (mm^−1^)	0.76
Crystal size (mm)	0.08 × 0.06 × 0.04

Data collection	
Diffractometer	Bruker APEXII
Absorption correction	Multi-scan (*SADABS*; Bruker, 2021 ▸)
*T*_min_, *T*_max_	0.673, 0.754
No. of measured, independent and observed [*I* > 2σ(*I*)] reflections	101717, 5548, 5338
*R*_int_	0.055
(sin θ/λ)_max_ (Å^−1^)	0.617

Refinement	
*R*[*F*^2^ > 2σ(*F*^2^)], *wR*(*F*^2^), *S*	0.041, 0.109, 1.07
No. of reflections	5548
No. of parameters	349
H-atom treatment	H-atom parameters constrained
Δρ_max_, Δρ_min_ (e Å^−3^)	0.34, −0.22
Absolute structure	Flack *x* determined using 2264 quotients [(*I*^+^) − (*I*^−^)]/[(*I*^+^) + (*I*^−^)] (Parsons *et al.*, 2013 ▸)
Absolute structure parameter	−0.02 (3)

Computer programs: *APEX2* (Bruker, 2021 ▸), *SAINT* (Bruker, 2021 ▸), *SORTAV* (Blessing, 1995 ▸), *SHELXT* (Sheldrick, 2015*a* ▸) and *SHELXL2018* (Sheldrick, 2015*b* ▸).

**Table 3:** Hydrogen-bond geometry (Å, °)

*D*—H⋯*A*	*D*—H	H⋯*A*	*D*⋯*A*	*D*—H⋯*A*
O7—H7⋯O8^i^	0.84	1.94	2.761 (3)	167
O8—H8⋯O2^ii^	0.84	2.10	2.895 (3)	158
C4—H4⋯O1^iii^	1.00	2.55	3.467 (3)	153
C13—H13*B*⋯O9^iv^	0.99	2.65	3.490 (3)	143
C7′—H7′⋯O6^v^	0.95	2.62	3.473 (3)	150

Symmetry codes: (i) 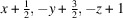; (ii) 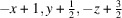; (iii) 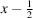, 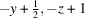; (iv) 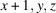; (v) 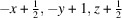.

## References

[bb1] Amagata, T., Rath, C., Rigot, J. F., Tarlov, N., Tenney, K., Valeriote, F. A. & Crews, P. (2003). *J. Med. Chem.* **46**, 4342–4350. 10.1021/jm030090t 13678412

[bb2] Blessing, R. H. (1995). *Acta Cryst.* A**51**, 33–38. 10.1107/s0108767394005726 7702794

[bb3] Bräse, S., Encinas, A., Keck, J. & Nising, C. F. (2009). *Chem. Rev.* **109**, 3903–3990. 10.1021/cr050001f 19534495

[bb4] Bruker (2021). *APEX4*, *SAINT* and *SADABS*. Bruker AXS Inc., Madison, Wisconsin, USA.

[bb5] Carvalho, M. de, Weich, H. & Abraham, W.-R. (2015). *Curr. Med. Chem.* **23**, 23–35. 10.2174/0929867323666151117121521 26572613

[bb6] Flack, H. D. (1983). *Acta Cryst.* A**39**, 876–881.

[bb7] Groom, C. R., Bruno, I. J., Lightfoot, M. P. & Ward, S. C. (2016). *Acta Cryst.* B**72**, 171–179. 10.1107/S2052520616003954 PMC4822653 27048719

[bb8] Grove, J. F. (2007). *Progress in the Chemistry of Organic Natural Products*, Vol. 88, pp. 63–130. Vienna: Springer Vienna.

[bb9] Jarvis, B. B., Comezoglu, S. N., Rao, M. M., Pena, N. B., Boettner, F. E., Williams, T. M., Forsyth, G. & Epling, B. (1987). *J. Org. Chem.* **52**, 45–56.

[bb10] Jarvis, B. B. & Mazzola, E. P. (1982). *Acc. Chem. Res.* **15**, 388–395.

[bb11] Jarvis, B. B., Midiwo, J. O., Flippen-Anderson, J. L. & Mazzola, E. P. (1982). *J. Nat. Prod.* **45**, 440–448.

[bb12] Jarvis, B. B. & Wang, S. (1999). *J. Nat. Prod.* **62**, 1284–1289. 10.1021/np990272j 10514314

[bb13] Jarvis, B. B., Wang, S. & Ammon, H. L. (1996). *J. Nat. Prod.* **59**, 254–261. 10.1021/np960078m 8882427

[bb14] McCormick, S. P., Stanley, A. M., Stover, N. A. & Alexander, N. J. (2011). *Toxins*, **3**, 802–814. 10.3390/toxins3070802 PMC3202860 22069741

[bb15] Parsons, S., Flack, H. D. & Wagner, T. (2013). *Acta Cryst.* B**69**, 249–259. 10.1107/S2052519213010014 PMC3661305 23719469

[bb16] Shank, R. A., Foroud, N. A., Hazendonk, P., Eudes, F. & Blackwell, B. A. (2011). *Toxins*, **3**, 1518–1553. 10.3390/toxins3121518 PMC3268455 22295175

[bb17] Sheldrick, G. M. (2015*a*). *Acta Cryst.* A**71**, 3–8.

[bb18] Sheldrick, G. M. (2015*b*). *Acta Cryst.* C**71**, 3–8.

[bb19] Sy-Cordero, A. A., Graf, T. N., Wani, M. C., Kroll, D. J., Pearce, C. J. & Oberlies, N. H. (2010). *J. Antibiot.* **63**, 539–544. 10.1038/ja.2010.77 PMC2946446 20648023

[bb20] Wu, Q., Wang, X., Nepovimova, E., Miron, A., Liu, Q., Wang, Y., Su, D., Yang, H., Li, L. & Kuca, K. (2017). *Arch. Toxicol.* **91**, 3737–3785. 10.1007/s00204-017-2118-3 29152681

[bb21] Zhu, M., Cen, Y., Ye, W., Li, S. & Zhang, W. (2020). *Toxins*, **12**, 417–433. 10.3390/toxins12060417 PMC7354583 32585939

