# Supplementary material for: Crystal structure of the cytotoxic macrocyclic trichothecene Isororidin A
Source: Acta Crystallogr C Struct Chem. 2024 Jul 10;80(Pt 8):407–11. doi: 10.1107/S2053229624006144 (PMC11299208; doi:10.1107/S2053229624006144)
Supplement: Supplementary file 4 [file c-80-00407-sup4.pdf]

Volume 80 (2024)

Supporting information for article:

Crystal structure of the cytotoxic macrocyclic trichothecene Isororidin A

Mostafa A. Asmaey, Dimitris A. Kalofolias, Maria-Despoina Charavgi, Ismail R. Abdel-Rahim, Evangelia D. Chrysina and Dennis Abatis

Computing details

Isororidin A at 293 K: Data collection: *CrysAlis PRO* 1.171.40.67a (Rigaku OD, 2019) (*Agilent Technologies, 2014*); cell refinement: *CrysAlis PRO* 1.171.40.67a (Rigaku OD, 2019) (*Agilent Technologies, 2014*) reduction: *CrysAlis PRO* 1.171.40.67a (Rigaku OD, 2019) (*Agilent Technologies, 2014*); program(s) used to solve structure: SHELXT (Sheldrick, 2015a); program(s) used to refine structure: *SHELXL2018*/3 (Sheldrick, 2015b).

Isororidin A at 100 K: Data collection: *APEX2* and *SAINT V8.40B* (Bruker AXS, 2021); cell refinement: *APEX4* and *SAINT V8.40B* (Bruker AXS, 2021) reduction: *APEX4* and *SAINT V8.40B* (Bruker AXS, 2021); integration software: *SORTAV* (Blessing, 1995), program(s) used to solve structure: SHELXT (Sheldrick, 2015a); program(s) used to refine structure: *SHELXL2018*/3 (Sheldrick, 2015b).

References

Agilent Technologies (2014). *Technol. UK Ltd, Yarnton, Oxford, UK.*

Table S1: Experimental details of Isororidin A crystal data collected at 293 K

Crystal data	
$C_{29}H_{40}O_9$	D_x = 1.222 Mg m^{-3}
M_r = 532.61	Cu *K*a radiation, l = 1.54180 Å
Orthorhombic, $P2_12_12_1$	Cell parameters from 3458 reflections
a = 9.302 (3) Å	q = 4.3–72.7°
b = 15.412 (6) Å	m = 0.74 mm^{-1}
c = 20.191 (8) Å	*T* = 293 K
V = 2894.6 (19) Å^3	Block, colorless
Z = 4	0.47 × 0.47 × 0.24 mm
F(000) = 1144	
Data collection	
SuperNova (Cu) X-ray Source diffractometer at Instruct-EL hub/INSPIRED, NHRF.	Absorption correction: analytical *CrysAlis PRO* 1.171.40.67a (Rigaku Oxford Diffraction, 2019) Analytical numeric absorption correction using a multifaceted crystal model based on expressions derived by R.C. Clark & J.S. Reid. (Clark, R. C. & Reid, J. S. (1995). Acta Cryst. A51, 887-897) Empirical absorption correction
Radiation source: micro-focus sealed X-ray tube	

	using spherical harmonics, implemented in SCALE3 ABSPACK scaling algorithm.
	T_{min} = 0.775, T_{max} = 0.878
	6984 measured reflections
	5037 independent reflections
	4285 reflections with $I > 2s(I)$
	R_{int} = 0.073
	q_{max} = 72.9°, q_{min} = 3.6°
	h = -6→11
	k = -16→18
	l = -24→24
Refinement	
Refinement on F^2	Hydrogen site location: inferred from neighbouring sites
Least-squares matrix: full	H-atom parameters constrained
$R[F^2 > 2\sigma(F^2)]$ = 0.086	$w = 1/[\sigma^2(F_o^2) + (0.2P)^2]$ where $P = (F_o^2 + 2F_c^2)/3$
$wR(F^2)$ = 0.265	$(\Delta/\sigma)_{max}$ < 0.001
S = 1.05	$\Delta\rho_{max}$ = 0.34 e Å^{-3}
5037 reflections	$\Delta\rho_{min}$ = -0.34 e Å^{-3}
349 parameters	Absolute structure: Classical Flack method preferred over Parsons because s.u. lower.
0 restraints	Absolute structure parameter: 0.4 (4)

Special details

Geometry. All esds (except the esd in the dihedral angle between two l.s. planes) are estimated using the full covariance matrix. The cell esds are taken into account individually in the estimation of esds in distances, angles and torsion angles; correlations between esds in cell parameters are only used when they are defined by crystal symmetry. An approximate (isotropic) treatment of cell esds is used for estimating esds involving l.s. planes.

Table S2: Experimental details of Isororidin A crystal data collected at 100 K

Crystal data	
$C_{29}H_{40}O_9$	D_x = 1.248 Mg m^{-3}
M_r = 532.61	Cu *K*a radiation, λ = 1.54178 Å
Orthorhombic, $P2_12_12_1$	Cell parameters from 9959 reflections
a = 9.271 (4) Å	θ = 4.4–74.9°
b = 15.224 (6) Å	μ = 0.76 mm^{-1}
c = 20.081 (8) Å	*T* = 100 K
V = 2834.0 (2) Å^3	Irregular plate, colourless
Z = 4	0.08 × 0.06 × 0.04 mm
F(000) = 1144	
Data collection	
Bruker APEX-II diffractometer	Absorption correction: multi-scan
Radiation source: Sealed x-ray tube	*SADABS2016/2 - Bruker AXS area detector scaling and absorption correction*
	T_{min} = 0.673, T_{max} = 0.754
	101717 measured reflections
	5548 independent reflections
	5338 reflections with $I > 2\sigma(I)$
	R_{int} = 0.055
	θ_{max} = 72.1°, θ_{min} = 3.6°
	h = -11→11
	k = -18→18
	l = -24→24
Refinement	
Refinement on F^2	Hydrogen site location: inferred from neighboring sites
Least-squares matrix: full	H-atom parameters constrained
$R[F^2 > 2\sigma(F^2)]$ = 0.041	$w = 1/[\sigma^2(F_o^2) + (0.0618P)^2 + 1.0952P]$ where $P = (F_o^2 + 2F_c^2)/3$
$wR(F^2)$ = 0.109	$(\Delta/\sigma)_{max}$ < 0.001

S = 1.07	$\Delta\rho_{max}$ = 0.34 e Å^{-3}
5548 reflections	$\Delta\rho_{min}$ = -0.22 e Å^{-3}
349 parameters	Absolute structure: Flack x determined using 2264 quotients [(I+)-(I-)]/[(I+)+(I-)] (Parsons, Flack and Wagner, Acta Cryst. B69 (2013) 249-259).

Special details

Geometry. All esds (except the esd in the dihedral angle between two l.s. planes) are estimated using the full covariance matrix. The cell esds are taken into account individually in the estimation of esds in distances, angles and torsion angles; correlations between esds in cell parameters are only used when they are defined by crystal symmetry. An approximate (isotropic) treatment of cell esds is used for estimating esds involving l.s. planes.

Table S3: Fractional atomic coordinates and isotropic or equivalent isotropic displacement parameters (Å^2) for Isororidin A measured at 100 K

	x	*y*	*z*	U_{iso}*/U_{eq}
O1	0.88908 (18)	0.27189 (12)	0.51273 (8)	0.0240 (4)
O2	0.9025 (2)	0.38349 (12)	0.67471 (9)	0.0272 (4)
O3	0.55743 (19)	0.51939 (11)	0.47181 (9)	0.0247 (4)
O4	0.1743 (2)	0.69785 (13)	0.64506 (9)	0.0308 (4)
O5	0.55825 (19)	0.34501 (11)	0.66449 (8)	0.0220 (4)
O6	0.3838 (2)	0.49608 (15)	0.39552 (11)	0.0424 (5)
O7	0.2988 (2)	0.66973 (14)	0.40501 (10)	0.0397 (5)
H7	0.337967	0.671580	0.367303	0.060*
O8	−0.0389 (2)	0.80771 (13)	0.70903 (10)	0.0361 (5)
H8	0.003475	0.841615	0.735599	0.054*
O9	0.37443 (19)	0.41830 (12)	0.61579 (9)	0.0269 (4)
C2	0.8665 (3)	0.27417 (17)	0.58343 (12)	0.0239 (5)
H2	0.941895	0.239374	0.607361	0.029*
C3	0.7140 (3)	0.24545 (15)	0.60435 (12)	0.0237 (5)
H3A	0.714192	0.222712	0.650543	0.028*

H3B	0.677134	0.199050	0.574293	0.028*
C4	0.6207 (3)	0.32912 (15)	0.59909 (11)	0.0198 (5)
H4	0.543332	0.321485	0.564896	0.024*
C5	0.7257 (2)	0.40513 (16)	0.57878 (12)	0.0189 (5)
C6	0.7370 (3)	0.40627 (15)	0.50005 (11)	0.0194 (5)
C7	0.8563 (3)	0.46869 (17)	0.47578 (13)	0.0255 (5)
H7A	0.835163	0.528884	0.491673	0.031*
H7B	0.949524	0.450303	0.495403	0.031*
C8	0.8701 (3)	0.46984 (18)	0.39997 (13)	0.0301 (6)
H8A	0.790742	0.505592	0.381129	0.036*
H8B	0.962284	0.498322	0.387684	0.036*
C9	0.8652 (3)	0.37965 (19)	0.36967 (12)	0.0280 (6)
C10	0.8205 (3)	0.31031 (17)	0.40345 (12)	0.0250 (5)
H10	0.818015	0.255442	0.380946	0.030*
C11	0.7731 (3)	0.31230 (16)	0.47532 (11)	0.0209 (5)
H11	0.684913	0.275005	0.480016	0.025*
C12	0.8689 (3)	0.36850 (17)	0.60468 (12)	0.0226 (5)
C13	0.9983 (3)	0.4175 (2)	0.62296 (13)	0.0289 (6)
H13A	0.998626	0.481667	0.614993	0.035*
H13B	1.092774	0.388245	0.616854	0.035*
C14	0.6885 (3)	0.49472 (15)	0.60854 (13)	0.0228 (5)
H14A	0.758586	0.538439	0.593183	0.034*
H14B	0.691533	0.491081	0.657245	0.034*
H14C	0.591501	0.512065	0.594273	0.034*
C15	0.5923 (3)	0.42707 (16)	0.46636 (12)	0.0221 (5)
H15A	0.597356	0.410455	0.418753	0.027*
H15B	0.515051	0.391867	0.487463	0.027*
C16	0.9125 (3)	0.3731 (2)	0.29815 (14)	0.0380 (7)
H16A	1.014581	0.389273	0.294656	0.057*

H16B	0.854565	0.413067	0.270815	0.057*
H16C	0.899321	0.312700	0.282417	0.057*
C1'	0.4428 (3)	0.54367 (19)	0.43490 (13)	0.0296 (6)
C2'	0.3983 (3)	0.63724 (18)	0.45204 (13)	0.0287 (6)
H2'	0.485817	0.675635	0.452173	0.034*
C3'	0.3294 (3)	0.63843 (18)	0.52177 (14)	0.0291 (6)
H3'	0.395944	0.606240	0.552451	0.035*
C4'	0.3149 (4)	0.7322 (2)	0.54813 (16)	0.0407 (7)
H4'A	0.399591	0.766598	0.533323	0.049*
H4'B	0.228023	0.759272	0.528101	0.049*
C5'	0.3034 (4)	0.7386 (2)	0.62343 (17)	0.0407 (7)
H5'A	0.387367	0.709419	0.644329	0.049*
H5'B	0.303563	0.801101	0.637090	0.049*
C6'	0.1495 (3)	0.69731 (18)	0.71470 (13)	0.0274 (5)
H6'	0.209471	0.744725	0.735249	0.033*
C7'	0.1896 (3)	0.61153 (17)	0.74684 (13)	0.0277 (5)
H7'	0.166965	0.605138	0.792725	0.033*
C8'	0.2536 (3)	0.54364 (17)	0.71731 (13)	0.0265 (5)
H8'	0.270664	0.545400	0.670677	0.032*
C9'	0.2979 (3)	0.46714 (17)	0.75504 (13)	0.0281 (5)
H9'	0.264277	0.463987	0.799677	0.034*
C10'	0.3807 (3)	0.40016 (17)	0.73433 (12)	0.0267 (5)
H10'	0.406954	0.356344	0.765766	0.032*
C11'	0.4333 (3)	0.39102 (15)	0.66526 (12)	0.0231 (5)
C12'	0.1877 (3)	0.5878 (2)	0.51995 (15)	0.0372 (6)
H12A	0.122060	0.615490	0.487947	0.056*
H12B	0.143561	0.588379	0.564304	0.056*
H12C	0.206171	0.526987	0.506428	0.056*
C13'	−0.0095 (3)	0.71915 (18)	0.72682 (13)	0.0298 (6)

H13'	−0.031032	0.711192	0.775237	0.036*
C14'	−0.1081 (3)	0.65997 (19)	0.68701 (15)	0.0334 (6)
H14D	−0.208572	0.677363	0.694432	0.050*
H14E	−0.094561	0.598944	0.701241	0.050*
H14F	−0.085000	0.665237	0.639549	0.050*

Table S4: Atomic displacement parameters (Å^2) for Isororidin A measured at 100 K

	U11	U22	U33	U12	U13	U23
O1	0.0212 (8)	0.0305 (9)	0.0202 (8)	0.0080 (7)	0.0008 (7)	0.0009 (7)
O2	0.0247 (9)	0.0368 (10)	0.0201 (8)	−0.0014 (8)	−0.0056 (7)	0.0005 (7)
O3	0.0238 (8)	0.0235 (8)	0.0268 (9)	0.0042 (7)	−0.0031 (7)	0.0025 (7)
O4	0.0297 (10)	0.0358 (10)	0.0268 (9)	−0.0051 (8)	0.0036 (8)	−0.0023 (8)
O5	0.0215 (8)	0.0251 (8)	0.0194 (8)	0.0011 (7)	0.0014 (7)	−0.0021 (7)
O6	0.0451 (12)	0.0461 (12)	0.0359 (11)	0.0143 (10)	−0.0180 (10)	−0.0071 (9)
O7	0.0404 (11)	0.0464 (12)	0.0324 (10)	0.0195 (10)	0.0088 (9)	0.0164 (9)
O8	0.0454 (12)	0.0303 (9)	0.0326 (10)	0.0084 (9)	−0.0132 (9)	−0.0085 (8)
O9	0.0213 (8)	0.0327 (9)	0.0268 (9)	0.0046 (7)	−0.0003 (8)	−0.0053 (8)
C2	0.0220 (12)	0.0292 (13)	0.0205 (11)	0.0070 (10)	−0.0013 (9)	0.0027 (10)
C3	0.0282 (12)	0.0215 (12)	0.0216 (11)	0.0034 (10)	0.0038 (10)	0.0021 (9)
C4	0.0208 (11)	0.0216 (11)	0.0171 (11)	0.0007 (9)	0.0011 (9)	−0.0025 (8)
C5	0.0165 (10)	0.0210 (11)	0.0192 (11)	0.0011 (9)	−0.0027 (9)	−0.0016 (9)
C6	0.0180 (11)	0.0217 (11)	0.0184 (11)	−0.0007 (9)	−0.0013 (9)	−0.0004 (9)
C7	0.0232 (12)	0.0268 (12)	0.0265 (13)	−0.0043 (10)	−0.0015 (10)	0.0020 (10)
C8	0.0286 (13)	0.0345 (14)	0.0272 (13)	−0.0061 (11)	0.0013 (11)	0.0074 (11)
C9	0.0228 (12)	0.0406 (15)	0.0206 (12)	0.0018 (11)	−0.0005 (10)	0.0004 (10)
C10	0.0237 (12)	0.0300 (12)	0.0212 (12)	0.0055 (11)	−0.0008 (10)	−0.0033 (10)
C11	0.0194 (11)	0.0237 (11)	0.0196 (11)	0.0023 (9)	0.0002 (9)	−0.0009 (9)
C12	0.0197 (11)	0.0314 (13)	0.0167 (11)	0.0022 (10)	−0.0012 (9)	0.0019 (9)
C13	0.0196 (11)	0.0413 (15)	0.0258 (12)	−0.0022 (11)	−0.0042 (10)	0.0028 (11)
C14	0.0221 (11)	0.0212 (11)	0.0252 (12)	−0.0005 (9)	−0.0014 (10)	−0.0034 (9)

C15	0.0211 (12)	0.0213 (11)	0.0240 (12)	0.0006 (9)	−0.0042 (9)	−0.0003 (9)
C16	0.0398 (17)	0.0503 (17)	0.0239 (13)	0.0020 (14)	0.0043 (12)	0.0043 (12)
C1'	0.0300 (13)	0.0345 (14)	0.0243 (13)	0.0043 (12)	0.0010 (11)	0.0051 (11)
C2'	0.0276 (13)	0.0294 (13)	0.0289 (13)	0.0047 (11)	0.0065 (10)	0.0065 (10)
C3'	0.0295 (13)	0.0306 (13)	0.0272 (13)	0.0022 (11)	0.0054 (11)	0.0037 (10)
C4'	0.0431 (17)	0.0292 (14)	0.0498 (18)	−0.0002 (14)	0.0197 (15)	0.0049 (13)
C5'	0.0394 (16)	0.0319 (15)	0.0506 (18)	−0.0101 (13)	0.0124 (14)	−0.0109 (13)
C6'	0.0306 (13)	0.0275 (12)	0.0242 (12)	−0.0018 (11)	−0.0019 (10)	−0.0061 (10)
C7'	0.0275 (13)	0.0318 (13)	0.0238 (12)	0.0003 (11)	−0.0022 (10)	−0.0052 (10)
C8'	0.0253 (12)	0.0294 (13)	0.0247 (12)	−0.0017 (10)	0.0008 (10)	−0.0056 (10)
C9'	0.0261 (12)	0.0326 (13)	0.0256 (12)	−0.0030 (11)	0.0057 (10)	−0.0040 (11)
C10'	0.0283 (12)	0.0273 (12)	0.0245 (12)	−0.0005 (11)	0.0048 (10)	−0.0003 (10)
C11'	0.0222 (12)	0.0211 (11)	0.0259 (12)	−0.0040 (10)	0.0024 (10)	−0.0039 (10)
C12'	0.0342 (15)	0.0449 (16)	0.0325 (14)	−0.0029 (14)	0.0073 (12)	0.0003 (12)
C13'	0.0366 (14)	0.0313 (14)	0.0215 (12)	0.0046 (12)	0.0012 (11)	−0.0049 (10)
C14'	0.0300 (14)	0.0328 (14)	0.0373 (15)	−0.0024 (12)	0.0009 (11)	−0.0014 (11)

Table S5: Geometric parameters (Å, º) for Isororidin A measured at 100K

O1—C2	1.436 (3)	C12—C13	1.460 (4)
O1—C11	1.449 (3)	C13—H13A	0.9900
O2—C12	1.458 (3)	C13—H13B	0.9900
O2—C13	1.462 (3)	C14—H14A	0.9800
O3—C1'	1.347 (3)	C14—H14B	0.9800
O3—C15	1.446 (3)	C14—H14C	0.9800
O4—C5'	1.416 (4)	C15—H15A	0.9900
O4—C6'	1.417 (3)	C15—H15B	0.9900
O5—C11'	1.354 (3)	C16—H16A	0.9800
O5—C4	1.456 (3)	C16—H16B	0.9800
O6—C1'	1.204 (4)	C16—H16C	0.9800
O7—C2'	1.409 (3)	C1'—C2'	1.523 (4)

O7—H7	0.8400	C2'—C3'	1.539 (4)
O8—C13'	1.421 (3)	C2'—H2'	1.0000
O8—H8	0.8400	C3'—C12'	1.524 (4)
O9—C11'	1.207 (3)	C3'—C4'	1.528 (4)
C2—C12	1.498 (4)	C3'—H3'	1.0000
C2—C3	1.538 (3)	C4'—C5'	1.519 (4)
C2—H2	1.0000	C4'—H4'A	0.9900
C3—C4	1.543 (3)	C4'—H4'B	0.9900
C3—H3A	0.9900	C5'—H5'A	0.9900
C3—H3B	0.9900	C5'—H5'B	0.9900
C4—C5	1.566 (3)	C6'—C7'	1.503 (4)
C4—H4	1.0000	C6'—C13'	1.530 (4)
C5—C14	1.528 (3)	C6'—H6'	1.0000
C5—C12	1.531 (3)	C7'—C8'	1.331 (4)
C5—C6	1.584 (3)	C7'—H7'	0.9500
C6—C15	1.535 (3)	C8'—C9'	1.449 (4)
C6—C7	1.538 (3)	C8'—H8'	0.9500
C6—C11	1.551 (3)	C9'—C10'	1.343 (4)
C7—C8	1.528 (4)	C9'—H9'	0.9500
C7—H7A	0.9900	C10'—C11'	1.477 (3)
C7—H7B	0.9900	C10'—H10'	0.9500
C8—C9	1.502 (4)	C12'—H12A	0.9800
C8—H8A	0.9900	C12'—H12B	0.9800
C8—H8B	0.9900	C12'—H12C	0.9800
C9—C10	1.321 (4)	C13'—C14'	1.512 (4)
C9—C16	1.505 (4)	C13'—H13'	1.0000
C10—C11	1.509 (3)	C14'—H14D	0.9800
C10—H10	0.9500	C14'—H14E	0.9800
C11—H11	1.0000	C14'—H14F	0.9800

C2—O1—C11	113.23 (17)	O3—C15—C6	111.26 (19)
C12—O2—C13	59.98 (16)	O3—C15—H15A	109.4
C1'—O3—C15	113.7 (2)	C6—C15—H15A	109.4
C5'—O4—C6'	116.2 (2)	O3—C15—H15B	109.4
C11'—O5—C4	115.90 (18)	C6—C15—H15B	109.4
C2'—O7—H7	109.5	H15A—C15—H15B	108.0
C13'—O8—H8	109.5	C9—C16—H16A	109.5
O1—C2—C12	107.62 (19)	C9—C16—H16B	109.5
O1—C2—C3	113.4 (2)	H16A—C16—H16B	109.5
C12—C2—C3	102.03 (19)	C9—C16—H16C	109.5
O1—C2—H2	111.1	H16A—C16—H16C	109.5
C12—C2—H2	111.1	H16B—C16—H16C	109.5
C3—C2—H2	111.1	O6—C1'—O3	123.7 (3)
C2—C3—C4	105.16 (19)	O6—C1'—C2'	126.0 (3)
C2—C3—H3A	110.7	O3—C1'—C2'	110.3 (2)
C4—C3—H3A	110.7	O7—C2'—C1'	110.7 (2)
C2—C3—H3B	110.7	O7—C2'—C3'	109.5 (2)
C4—C3—H3B	110.7	C1'—C2'—C3'	109.2 (2)
H3A—C3—H3B	108.8	O7—C2'—H2'	109.1
O5—C4—C3	107.36 (18)	C1'—C2'—H2'	109.1
O5—C4—C5	111.07 (18)	C3'—C2'—H2'	109.1
C3—C4—C5	106.24 (19)	C12'—C3'—C4'	113.9 (2)
O5—C4—H4	110.7	C12'—C3'—C2'	109.3 (2)
C3—C4—H4	110.7	C4'—C3'—C2'	111.3 (2)
C5—C4—H4	110.7	C12'—C3'—H3'	107.4
C14—C5—C12	112.8 (2)	C4'—C3'—H3'	107.4
C14—C5—C4	114.66 (19)	C2'—C3'—H3'	107.4
C12—C5—C4	100.44 (19)	C5'—C4'—C3'	114.3 (2)
C14—C5—C6	113.28 (19)	C5'—C4'—H4'A	108.7

C12—C5—C6	106.61 (18)	C3'—C4'—H4'A	108.7
C4—C5—C6	107.99 (18)	C5'—C4'—H4'B	108.7
C15—C6—C7	111.19 (19)	C3'—C4'—H4'B	108.7
C15—C6—C11	103.77 (18)	H4'A—C4'—H4'B	107.6
C7—C6—C11	108.23 (19)	O4—C5'—C4'	109.7 (3)
C15—C6—C5	112.62 (19)	O4—C5'—H5'A	109.7
C7—C6—C5	111.73 (19)	C4'—C5'—H5'A	109.7
C11—C6—C5	108.88 (18)	O4—C5'—H5'B	109.7
C8—C7—C6	112.5 (2)	C4'—C5'—H5'B	109.7
C8—C7—H7A	109.1	H5'A—C5'—H5'B	108.2
C6—C7—H7A	109.1	O4—C6'—C7'	112.9 (2)
C8—C7—H7B	109.1	O4—C6'—C13'	108.2 (2)
C6—C7—H7B	109.1	C7'—C6'—C13'	111.0 (2)
H7A—C7—H7B	107.8	O4—C6'—H6'	108.2
C9—C8—C7	113.0 (2)	C7'—C6'—H6'	108.2
C9—C8—H8A	109.0	C13'—C6'—H6'	108.2
C7—C8—H8A	109.0	C8'—C7'—C6'	126.4 (3)
C9—C8—H8B	109.0	C8'—C7'—H7'	116.8
C7—C8—H8B	109.0	C6'—C7'—H7'	116.8
H8A—C8—H8B	107.8	C7'—C8'—C9'	121.2 (2)
C10—C9—C8	122.1 (2)	C7'—C8'—H8'	119.4
C10—C9—C16	121.9 (3)	C9'—C8'—H8'	119.4
C8—C9—C16	116.0 (2)	C10'—C9'—C8'	127.6 (2)
C9—C10—C11	124.5 (2)	C10'—C9'—H9'	116.2
C9—C10—H10	117.8	C8'—C9'—H9'	116.2
C11—C10—H10	117.8	C9'—C10'—C11'	123.5 (2)
O1—C11—C10	105.75 (18)	C9'—C10'—H10'	118.3
O1—C11—C6	112.71 (18)	C11'—C10'—H10'	118.3
C10—C11—C6	112.8 (2)	O9—C11'—O5	123.7 (2)

O1—C11—H11	108.5	O9—C11'—C10'	126.3 (2)
C10—C11—H11	108.5	O5—C11'—C10'	110.0 (2)
C6—C11—H11	108.5	C3'—C12'—H12A	109.5
O2—C12—C13	60.13 (16)	C3'—C12'—H12B	109.5
O2—C12—C2	115.3 (2)	H12A—C12'—H12B	109.5
C13—C12—C2	125.0 (2)	C3'—C12'—H12C	109.5
O2—C12—C5	117.1 (2)	H12A—C12'—H12C	109.5
C13—C12—C5	127.7 (2)	H12B—C12'—H12C	109.5
C2—C12—C5	103.9 (2)	O8—C13'—C14'	108.4 (2)
C12—C13—O2	59.90 (15)	O8—C13'—C6'	110.6 (2)
C12—C13—H13A	117.8	C14'—C13'—C6'	111.6 (2)
O2—C13—H13A	117.8	O8—C13'—H13'	108.7
C12—C13—H13B	117.8	C14'—C13'—H13'	108.7
O2—C13—H13B	117.8	C6'—C13'—H13'	108.7
H13A—C13—H13B	114.9	C13'—C14'—H14D	109.5
C5—C14—H14A	109.5	C13'—C14'—H14E	109.5
C5—C14—H14B	109.5	H14D—C14'—H14E	109.5
H14A—C14—H14B	109.5	C13'—C14'—H14F	109.5
C5—C14—H14C	109.5	H14D—C14'—H14F	109.5
H14A—C14—H14C	109.5	H14E—C14'—H14F	109.5
H14B—C14—H14C	109.5		
C11—O1—C2—C12	−65.2 (2)	O1—C2—C12—C5	72.8 (2)
C11—O1—C2—C3	46.9 (3)	C3—C2—C12—C5	−46.8 (2)
O1—C2—C3—C4	−85.5 (2)	C14—C5—C12—O2	38.1 (3)
C12—C2—C3—C4	30.0 (2)	C4—C5—C12—O2	−84.4 (2)
C11'—O5—C4—C3	−154.6 (2)	C6—C5—C12—O2	163.10 (19)
C11'—O5—C4—C5	89.7 (2)	C14—C5—C12—C13	−33.7 (3)
C2—C3—C4—O5	−121.9 (2)	C4—C5—C12—C13	−156.3 (2)
C2—C3—C4—C5	−3.0 (2)	C6—C5—C12—C13	91.2 (3)

O5—C4—C5—C14	−29.1 (3)	C14—C5—C12—C2	166.5 (2)
C3—C4—C5—C14	−145.6 (2)	C4—C5—C12—C2	44.0 (2)
O5—C4—C5—C12	92.1 (2)	C6—C5—C12—C2	−68.5 (2)
C3—C4—C5—C12	−24.3 (2)	C2—C12—C13—O2	−101.5 (3)
O5—C4—C5—C6	−156.44 (18)	C5—C12—C13—O2	102.7 (3)
C3—C4—C5—C6	87.1 (2)	C1'—O3—C15—C6	170.8 (2)
C14—C5—C6—C15	−64.8 (3)	C7—C6—C15—O3	−51.0 (3)
C12—C5—C6—C15	170.5 (2)	C11—C6—C15—O3	−167.16 (18)
C4—C5—C6—C15	63.3 (2)	C5—C6—C15—O3	75.3 (2)
C14—C5—C6—C7	61.2 (3)	C15—O3—C1'—O6	−7.6 (4)
C12—C5—C6—C7	−63.5 (2)	C15—O3—C1'—C2'	170.7 (2)
C4—C5—C6—C7	−170.71 (19)	O6—C1'—C2'—O7	−13.8 (4)
C14—C5—C6—C11	−179.32 (19)	O3—C1'—C2'—O7	168.0 (2)
C12—C5—C6—C11	56.0 (2)	O6—C1'—C2'—C3'	106.9 (3)
C4—C5—C6—C11	−51.2 (2)	O3—C1'—C2'—C3'	−71.3 (3)
C15—C6—C7—C8	−53.9 (3)	O7—C2'—C3'—C12'	54.8 (3)
C11—C6—C7—C8	59.4 (3)	C1'—C2'—C3'—C12'	−66.6 (3)
C5—C6—C7—C8	179.3 (2)	O7—C2'—C3'—C4'	−71.8 (3)
C6—C7—C8—C9	−44.4 (3)	C1'—C2'—C3'—C4'	166.8 (3)
C7—C8—C9—C10	14.6 (4)	C12'—C3'—C4'—C5'	78.2 (4)
C7—C8—C9—C16	−166.1 (2)	C2'—C3'—C4'—C5'	−157.8 (3)
C8—C9—C10—C11	−1.4 (4)	C6'—O4—C5'—C4'	179.0 (2)
C16—C9—C10—C11	179.3 (2)	C3'—C4'—C5'—O4	−64.7 (4)
C2—O1—C11—C10	175.6 (2)	C5'—O4—C6'—C7'	−99.2 (3)
C2—O1—C11—C6	51.9 (3)	C5'—O4—C6'—C13'	137.6 (2)
C9—C10—C11—O1	−106.0 (3)	O4—C6'—C7'—C8'	5.8 (4)
C9—C10—C11—C6	17.6 (3)	C13'—C6'—C7'—C8'	127.4 (3)
C15—C6—C11—O1	−166.89 (18)	C6'—C7'—C8'—C9'	174.7 (2)
C7—C6—C11—O1	74.9 (2)	C7'—C8'—C9'—C10'	−169.7 (3)

C5—C6—C11—O1	−46.7 (2)	C8'—C9'—C10'—C11'	−4.7 (4)
C15—C6—C11—C10	73.4 (2)	C4—O5—C11'—O9	−0.1 (3)
C7—C6—C11—C10	−44.8 (3)	C4—O5—C11'—C10'	178.57 (19)
C5—C6—C11—C10	−166.4 (2)	C9'—C10'—C11'—O9	−28.5 (4)
C13—O2—C12—C2	117.4 (3)	C9'—C10'—C11'—O5	152.8 (2)
C13—O2—C12—C5	−119.9 (3)	O4—C6'—C13'—O8	−68.1 (3)
O1—C2—C12—O2	−157.67 (19)	C7'—C6'—C13'—O8	167.6 (2)
C3—C2—C12—O2	82.7 (2)	O4—C6'—C13'—C14'	52.7 (3)
O1—C2—C12—C13	−87.6 (3)	C7'—C6'—C13'—C14'	−71.6 (3)
C3—C2—C12—C13	152.8 (2)		

Table S6: Hydrogen-bond geometry (Å, º) for Isororidin A measured at 100 K

D—H···*A*	*D*—H	H···*A*	*D*···*A*	*D*—H···*A*
O7—H7···O8^{i}	0.84	1.94	2.761 (3)	166.7
O8—H8···O2^{ii}	0.84	2.10	2.895 (3)	157.7
C4—H4···O1^{iii}	1.00	2.55	3.467 (3)	152.6
C13—H13B···O9^{iv}	0.99	2.65	3.490 (3)	142.6
C7'—H7'···O6^{v}	0.95	2.62	3.473 (3)	149.8

Symmetry codes: (i) *x*+1/2, −*y*+3/2, −*z*+1; (ii) −*x*+1, *y*+1/2, −*z*+3/2; (iii) *x*−1/2, −*y*+1/2, −*z*+1; (iv) *x*+1, *y*, *z*; (v) −*x*+1/2, −*y*+1, *z*+1/2.

Table S7: Torsion angles (°) of Isororidin A measured at 100 K *vs* the corresponding torsion angles in the Roridin A structure by Jarvis *et. al*. (1982*b*) as discussed in the manuscript.

Isororidin A		Roridin A (Jarvis *et al*., 1982*b*)	
C5-C6-C11-O1	-46.7 (2)°	C4-C5-C10-O1	-41.5°
O4-C6'-C13'-O8	-68.1 (3)°	O4-C21-C28-O8	-174.8°
C7'-C6'-C13'-O8	167.6 (2)°	C22-C21-C28-O8	63.0°
O7-C2'-C3'-C4'	-71.8 (3)°	O7-C17-C18-C19	-53.4°
O7-C2'-C3'-C12'	54.8 (3)°	O7-C17-C18-C27	72.0°
O9-C11'-C10'-C9'	-28.6 (4)°	O9-C26-C25-C24	-10.9°
O5-C11'-C10'-C9'	152.8 (2)°	O5-C26-C25-C24	169.0°
C7'-C6'-C13'-C14'	-71.6 (3)°	C22-C21-C28-C29	-60.8°
O4-C6'-C13'-C14'	52.7 (3)°	O4-C21-C28-C29	61.9°
O6-C1'-C2'-C3'	106.9 (3)°	O6-C16-C17-C18	114.3°
O3-C1'-C2'-O7	168.0 (2)°	O3-C16-C17-O7	172.8°
O6-C1'-C2'-O7	-13.7 (4)°	O6-C16-C17-O7	-9.6°
C15-O3-C1'-C2'	170.7 (2)°	C14-O3-C16-C17	166.8°
C15-O3-C1'-O6	-7.61 (4)°	C14-O3-C16-O6	-10.8°

Table S8: Carbon atoms numbering followed in the Roridin A structure deposited by Jarvis *et al*. (1982*b*) *vs* Isororidin A

Roridin A (Jarvis *et al*., 1982*b*)	Isororidin A	Roridin A (Jarvis *et al*., 1982*b*)	Isororidin A
C1	C2	C16	C1'
C2	C3	C17	C2'
C3	C4	C18	C3'
C4	C5	C19	C4'
C5	C6	C20	C5'
C6	C7	C21	C6'
C7	C8	C22	C7'
C8	C9	C23	C8'
C9	C10	C24	C9'
C10	C11	C25	C10'
C11	C12	C26	C11'
C12	C13	C27	C12'
C13	C14	C28	C13'
C14	C15	C29	C14'
C15	C16		

Analytical Data

The molecular formula of Isororidin A ($C_{29}H_{40}O_9$) was deduced from positive HR-ESI-MS in which we detected a pseudomolecular ion peak at 550.2963 amu which corresponds to $[M+NH_4]^+$ (calculated 550.3011 amu, Δm = -8.7ppm).

HR-ESI-MS was measured on a Q-Exactive Orbitrap platform (Thermo Fisher Scientific, San Jose, CA, USA) connected to a Dionex Ultimate 3000 UHPLC system (Thermo Scientific™Dionex™, Sunnyvale, CA, USA) was employed. A Hypersil Gold UPLC C18 (2.1×150 mm, 1.9µm) reversed phase column (Thermo Fisher Scientific, San Jose, CA, USA) was used.

For positive ionization mode, the mobile phases were water/methanol 90/10 (solvent A) and methanol (solvent B) both amended with 5mM ammonium formate and 0.01% formic acid. The adopted gradient elution program started with 1% B with a flow rate of 0.2 mL min^{-1} for 1 min and it increased

to 39 % in 2 min (flow rate 0.2 mL min^{-1}), and then to 99.9 % (flow rate 0.4 mL min^{-1}) in the following 11 min. Then it kept constant for 2 min (flow rate 0.48 mL min^{-1}), then initial conditions were restored within 0.1 min, kept for 3 min and then the flow rate decreased to 0.2 mL min^{-1}. The injection volume was set up to 5 µL.

After a series of open-column chromatographic separations, the final semi-preparative HPLC purification was performed on a C18 column (250mm × 10mm × 5µm, Dr. Maisch GmbH, Germany), using a linear gradient elution scheme of MeOH/deionized water. HPLC system consisted of an ECOM ECP2010 pump, coupled with an ECB2004 gradient box with degasser (ECOM, Prague, Czech Rep.), a MISTRAL oven (Spark Holland, Emmen, Holland), an ALIAS autosampler (Spark Holland, Emmen, Holland), an ECOM ECDA2800 Diode Array Detector (DAD) and an ECF 200 fraction collector (ECOM, Prague, Czech Rep.).

NMR spectra were recorded at 298º K on a Bruker Avance III 600 spectrometer (600.23 MHz for ^{1}H, 150.93 MHz for ^{13}C) equipped with a PABBI inverse detection probe with a z-axis gradient coil and a B-ACS 60 autosampler and on a Bruker DRX 400 spectrometer (400.13 MHz for ^{1}H, 100.61 MHz for ^{13}C) equipped with a PABBI inverse detection probe with a z-axis gradient coil.

All 1D and 2D spectra were obtained using the standard Bruker software. Residual solvent signals were used as internal standards (reference signal) $CHCl_3$ (δH 7.26 ppm and δc 77.0) and CHD_2OD (δH 3.31 ppm and δC 49.0 ppm). The observed chemical shift (δ) values were given in ppm and the coupling constants (*J*) in Hz.

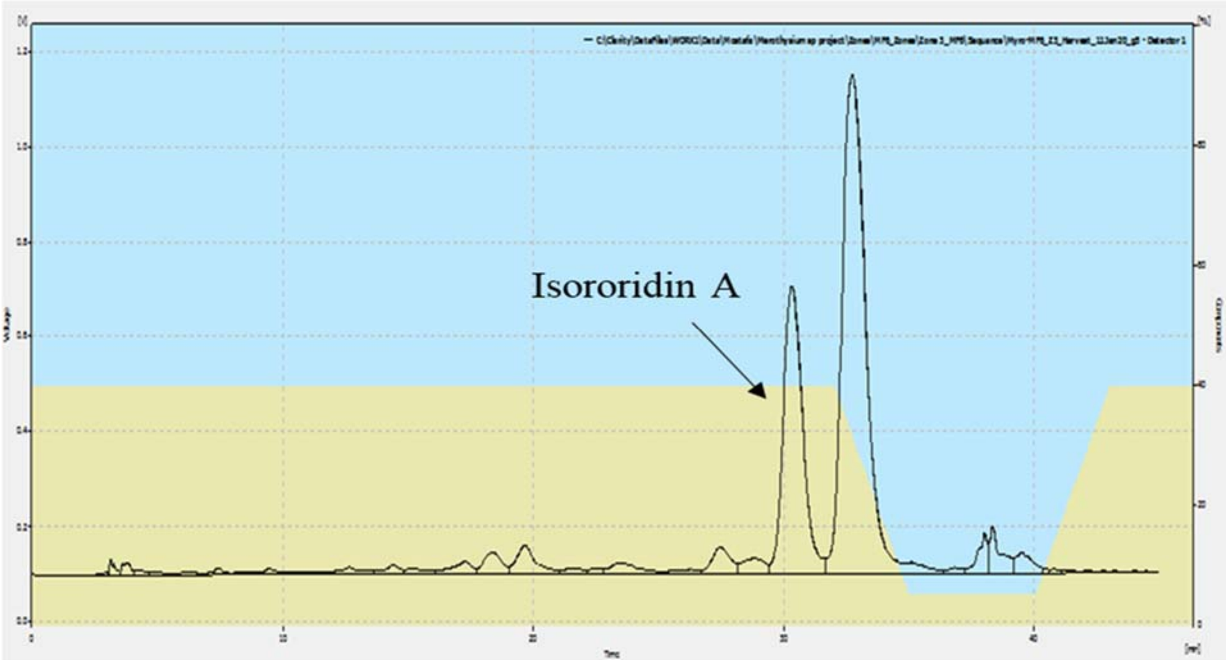

Figure S1: HPLC using semiprep. C18 column and eluted with H_2O/MeOH.

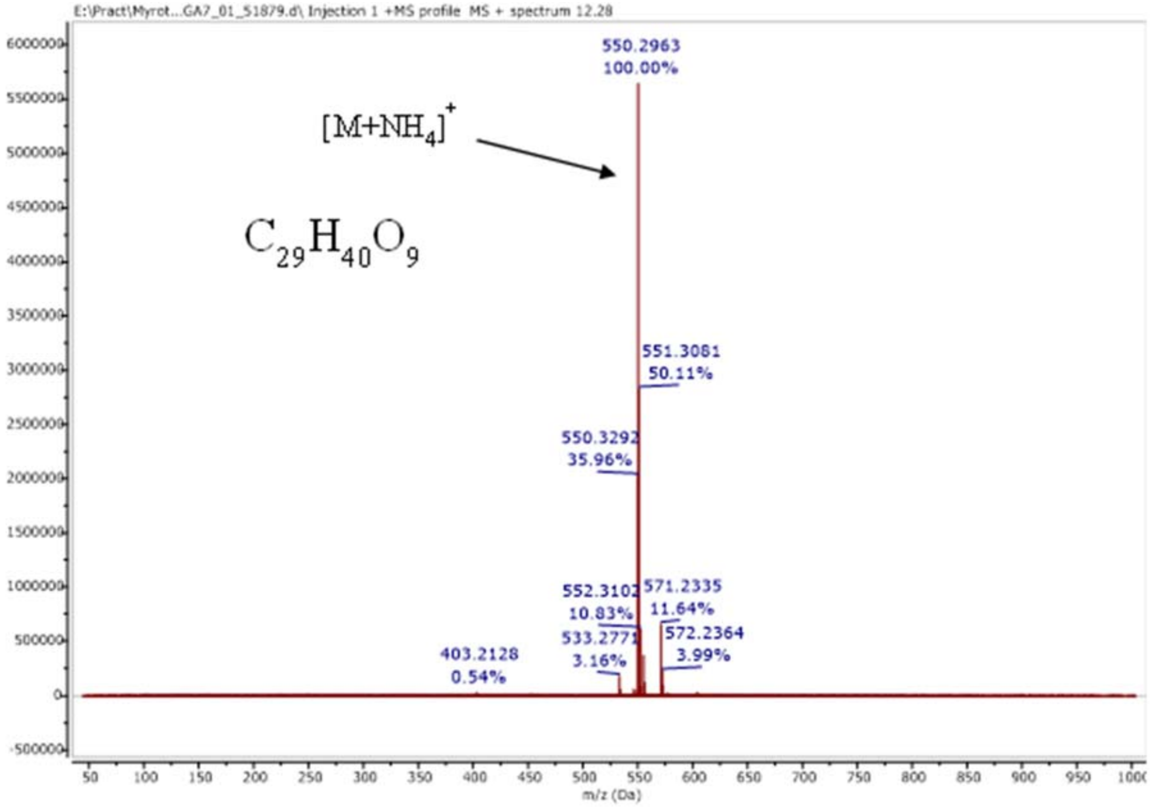

Figure S2: (+)HR-ESI Mass spectrum of Isororidin A.

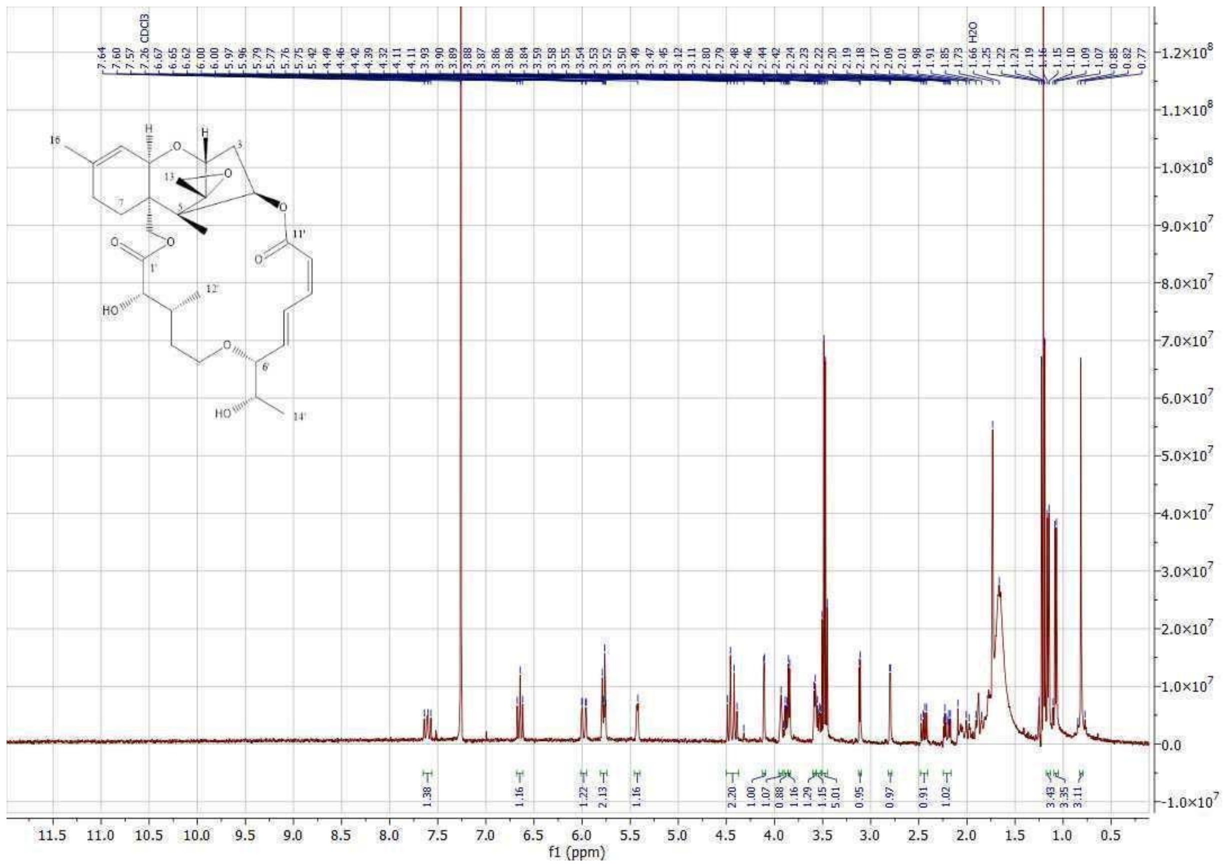

Figure S3: ^{1}H NMR spectrum of Isororidin A ($CDCl_3$)

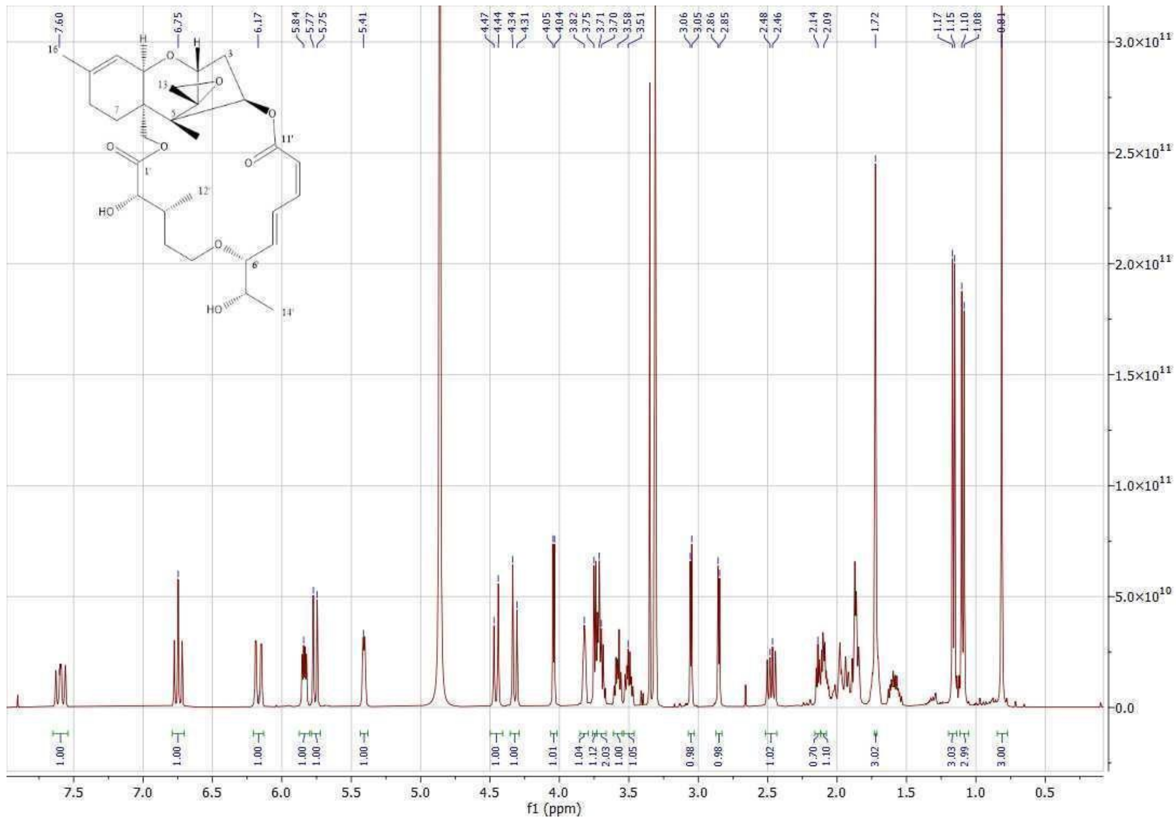

Figure S4: ^{1}H NMR spectrum of Isororidin A (MeOD-d_4).

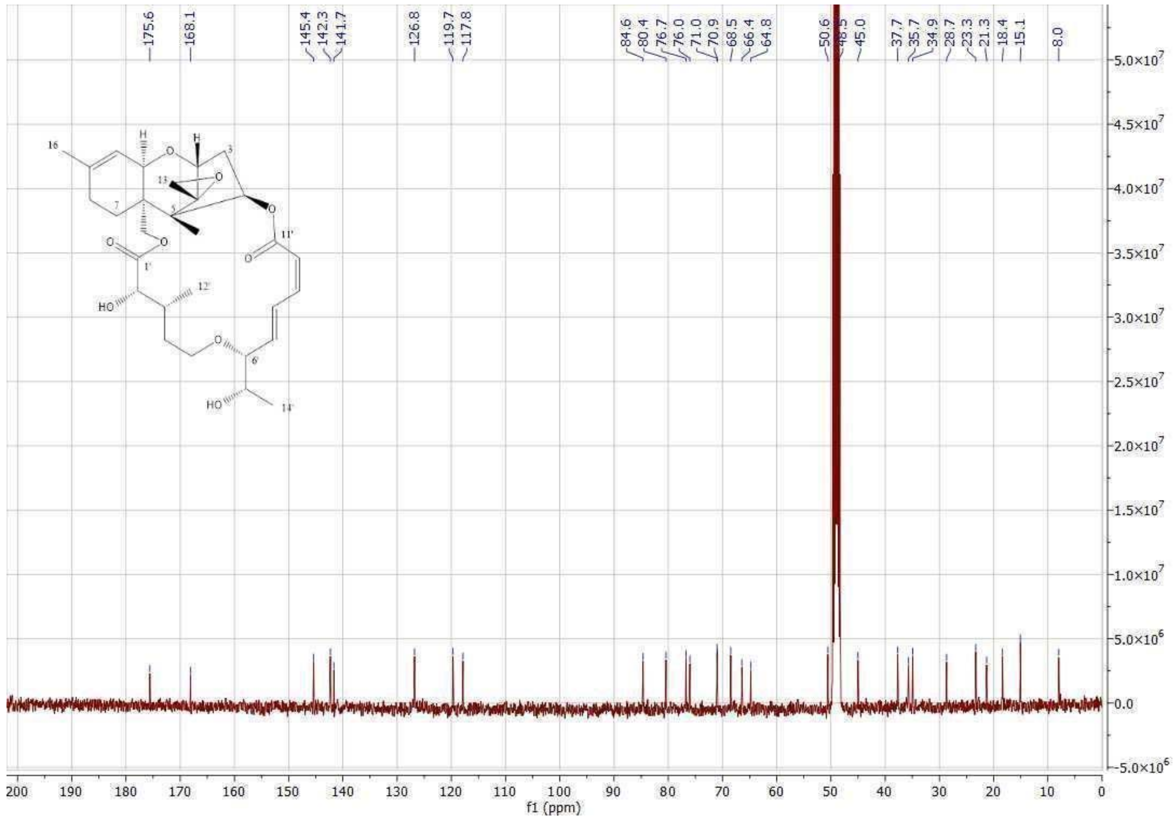

Figure S5: ^{13}C NMR spectrum of Isororidin A (MeOD-d_4).

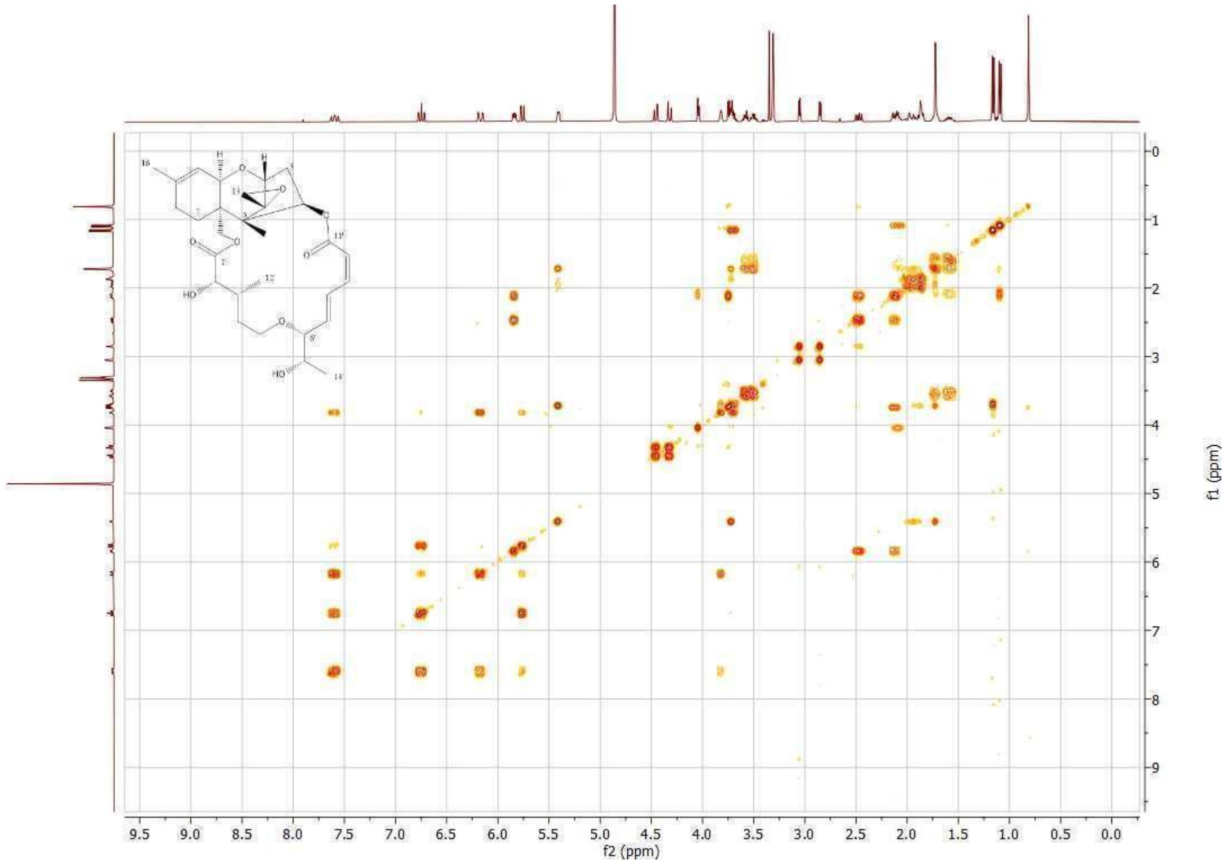

Figure S6: ^{1}H-^{1}H COSY spectrum of Isororidin A (MeOD-d_4).

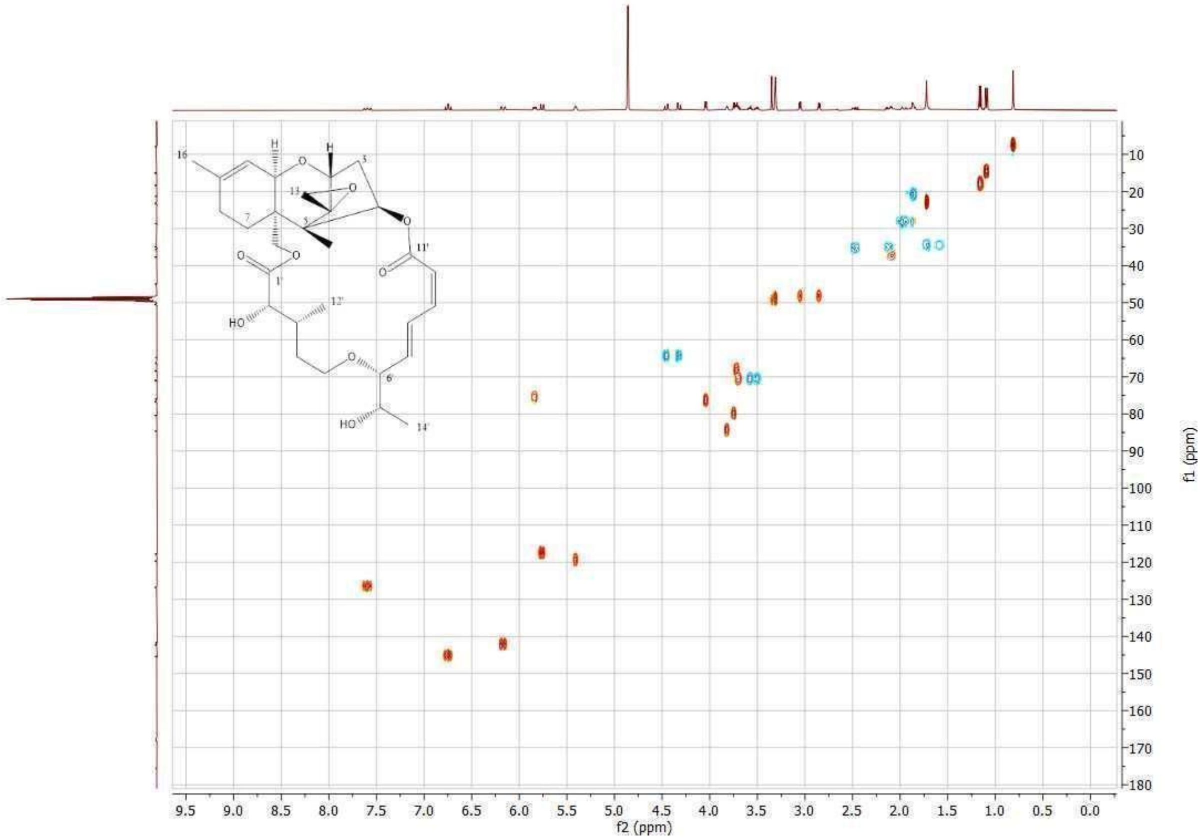

Figure S7: HSQC-DEPT spectrum of Isororidin A (MeOD-d_4).

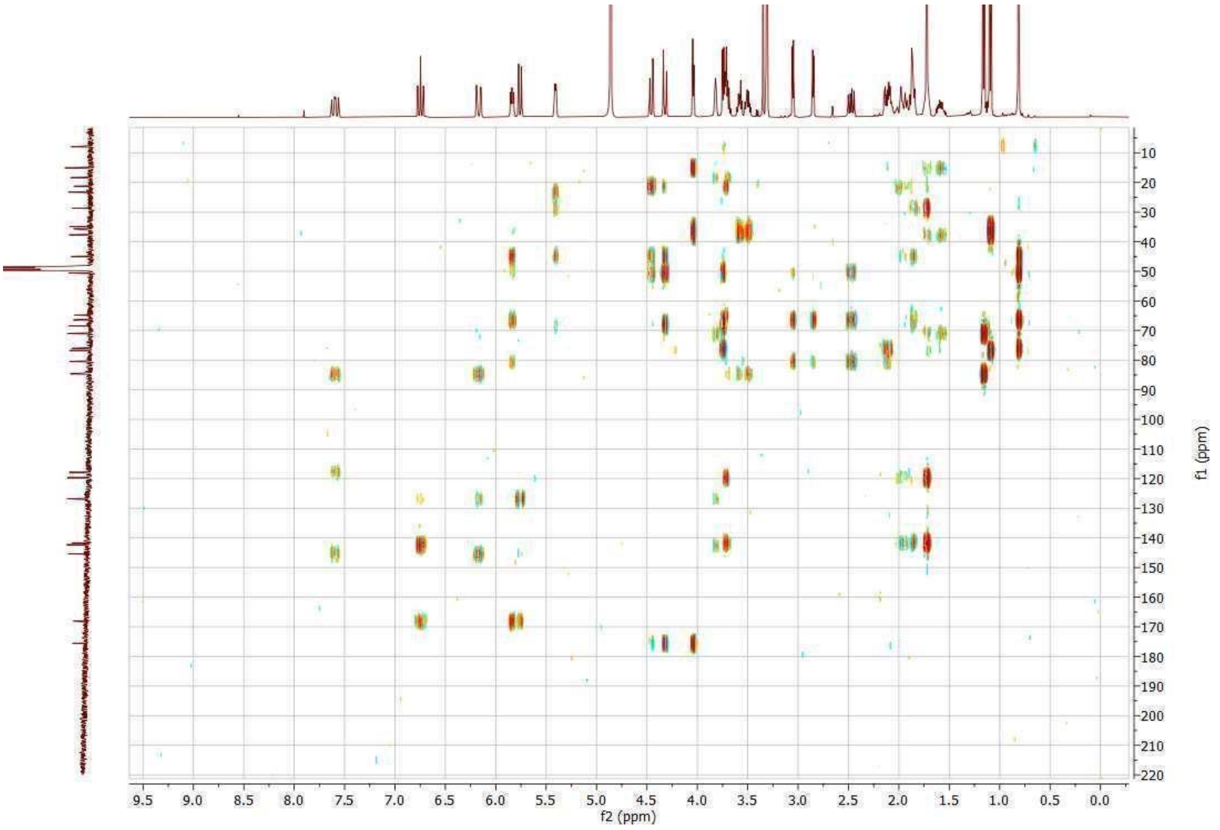

Figure S8: HMBC spectrum of Isororidin A (MeOD-d_4).

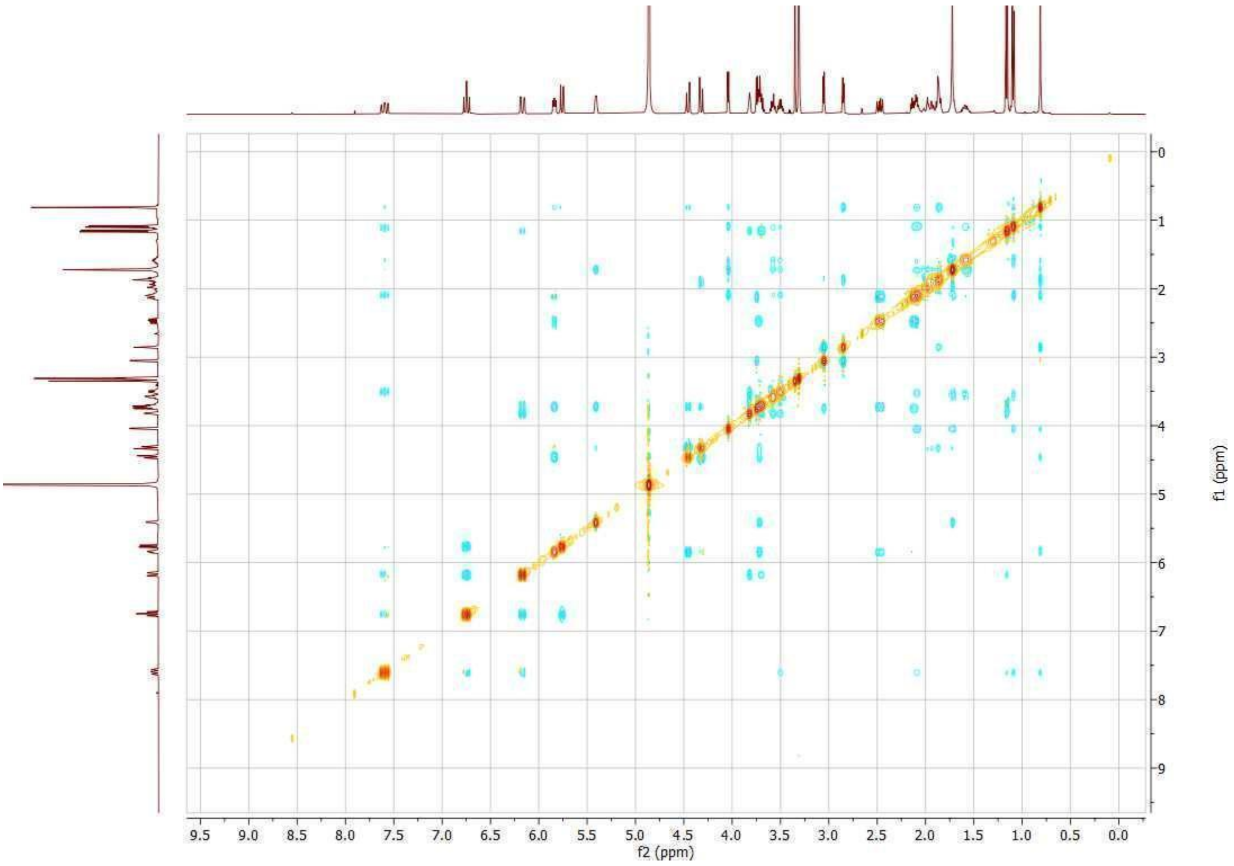

Figure S9: NOE spectrum of Isororidin A (MeOD-d_4).
